# Interactions of obesity, body shape, diabetes and sex steroids with respect to prostate cancer risk in the UK Biobank cohort

**DOI:** 10.1002/cam4.6918

**Published:** 2024-01-17

**Authors:** Sofia Christakoudi, Konstantinos K. Tsilidis, Evangelos Evangelou, Elio Riboli

**Affiliations:** ^1^ Department of Epidemiology and Biostatistics School of Public Health, Imperial College London London UK; ^2^ Department of Inflammation Biology School of Immunology and Microbial Sciences, King's College London London UK; ^3^ Department of Hygiene and Epidemiology University of Ioannina School of Medicine Ioannina Greece

**Keywords:** cancer prevention, cancer risk factors, epidemiology, prostate cancer

## Abstract

**Background:**

Obesity and diabetes are associated inversely with low‐grade prostate cancer risk and affect steroid hormone synthesis but whether they modify each other's impact on prostate cancer risk remains unknown.

**Methods:**

We examined the independent associations of diabetes, body mass index (BMI), ‘a body shape index’ (ABSI), hip index (HI), circulating testosterone, sex hormone binding globulin (SHBG) (per one standard deviation increase) and oestradiol ≥175 pmol/L with total prostate cancer risk using multivariable Cox proportional hazards models for UK Biobank men. We evaluated multiplicative interactions (*p*_MI_) and additive interactions (relative excess risk from interaction (*p*_RERI_), attributable proportion (*p*_AR_), synergy index (*p*_SI_)) with obese (BMI ≥30 kg/m^2^) and diabetes.

**Results:**

During a mean follow‐up of 10.3 years, 9417 incident prostate cancers were diagnosed in 195,813 men. Diabetes and BMI were associated more strongly inversely with prostate cancer risk when occurring together (*p*_MI_ = 0.0003, *p*_RERI_ = 0.032, *p*_AP_ = 0.020, *p*_SI_ = 0.002). ABSI was associated positively in obese men (HR = 1.081; 95% CI = 1.030–1.135) and men with diabetes (HR = 1.114; 95% CI = 1.021–1.216). The inverse associations with obesity and diabetes were attenuated for high‐ABSI ≥79.8 (*p*_MI_ = 0.022, *p*_RERI_ = 0.008, *p*_AP_ = 0.005, *p*_SI_ <0.0001 obesity; *p*_MI_ = 0.017, *p*_RERI_ = 0.047, *p*_AP_ = 0.025, *p*_SI_ = 0.0005 diabetes). HI was associated inversely in men overall (HR = 0.967; 95% CI = 0.947–0.988). Free testosterone (FT) was associated most strongly positively in normal weight men (HR = 1.098; 95% CI = 1.045–1.153) and men with diabetes (HR = 1.189; 95% CI = 1.081–1.308). Oestradiol was associated inversely in obese men (HR = 0.805; 95% CI = 0.682–0.951). The inverse association with obesity was stronger for high‐FT ≥243 pmol/L (*p*_RERI_ = 0.040, *p*_AP_ = 0.031, *p*_SI_ = 0.002) and high‐oestradiol (*p*_RERI_ = 0.030, *p*_AP_ = 0.012, *p*_SI_ <0.0001). The inverse association with diabetes was attenuated for high‐FT (*p*_MI_ = 0.008, *p*_RERI_ = 0.015, *p*_AP_ = 0.009, p_SI_ = 0.0006). SHBG was associated inversely in men overall (HR = 0.918; 95% CI = 0.895–0.941), more strongly for high‐HI ≥49.1 (*p*_MI_ = 0.024).

**Conclusions:**

Obesity and diabetes showed synergistic inverse associations with prostate cancer risk, likely involving testosterone reduction for diabetes and oestrogen generation for obesity, which were attenuated for high‐ABSI. HI and SHBG showed synergistic inverse associations with prostate cancer risk.

## Graphical abstract

Using UK Biobank data, we have shown synergistic inverse associations of obesity and diabetes with total prostate cancer risk, likely involving testosterone reduction for diabetes and oestrogen generation for obesity, which were attenuated in men with large waist size. Hip size and sex hormone binding globulin showed synergistic inverse associations with total prostate cancer risk.

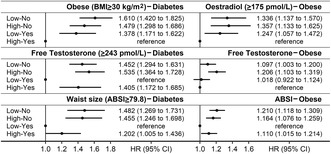

## 1 INTRODUCTION

Contrary to the conventional expectation that obesity and diabetes are associated with higher risk of cancer,^1^, ^2^ they are both associated with lower risk of low‐grade prostate cancer.^3^, ^4^ Given that obesity is associated with higher risk of diabetes, a question arises whether diabetes development is required for obesity to influence prostate cancer risk. Further, low‐grade prostate cancer is usually androgen‐dependent^5^ and both obesity and diabetes are associated with lower androgen levels.^6^, ^7^ A question, therefore, arises whether androgen suppression is involved in the inverse associations of obesity and diabetes with prostate cancer risk. Furthermore, obesity is associated with higher oestrogen levels,^6^ raising the question of oestrogen involvement in prostate cancer risk.

Absolute abdominal size evaluated with waist circumference (WC) is associated strongly positively with overall size evaluated with body mass index (BMI) and both reflect general obesity.^8^ Correspondingly, both WC and BMI have been associated inversely with prostate cancer risk in UK Biobank.^9^ Relative abdominal size, however, evaluated with ‘a body shape index’ (ABSI), has not been associated with prostate cancer risk in men overall,^8^, ^10^ despite an inverse association of ABSI with circulating total testosterone (TT) and free testosterone (FT).^6^ This is not paradoxical, because WC and ABSI define abdominal obesity differently. While WC is a better measure of abdominal fat quantity compared to BMI, which reflects overall fat quantity, ABSI reflects fat distribution.^11^ ABSI compares the abdominal size of a given individual with the average abdominal size of individuals with the same BMI and height and is thus uncorrelated with BMI^12^ and complements rather than replaces BMI. As the factors contributing to fat accumulation can differ from the factors contributing to fat distribution, it is important to clarify the association of ABSI with prostate cancer risk.

Hip circumference (HC), similarly to WC, reflects fat accumulation and is correlated strongly positively with BMI,^8^ while hip index (HI), in analogy to ABSI, reflects fat distribution.^11^ HI compares hip size of a given individual with the average hip size of individuals with the same BMI and height and is thus uncorrelated with BMI.^13^ Our previous cancer‐wide study in UK Biobank suggested an inverse association of HI with prostate cancer risk.^8^ Clarifying associations with HI is important because aromatase levels are highest in gluteofemoral adipose tissue^14^ and, correspondingly, circulating oestrogen levels are higher for higher HI.^6^

In this study, we used data from UK Biobank with additional follow‐up time compared to our previous study,^8^ to clarify the relationships between BMI, ABSI, HI, diabetes, circulating sex steroids, sex steroid binding globulin (SHBG) and prostate cancer risk in men overall and according to BMI categories and diabetes status.

## 2 METHODS

### 2.1 Study population

UK Biobank has recruited and is following‐up half a million participants aged 40–70 years at baseline (between 2006 and 2010), living within 40 km of an assessment centre in England, Scotland and Wales, and registered with the National Health Service.^15^ We restricted this study to men with self‐reported white ancestry, as other ethnic groups were limited. We additionally excluded 33,243 (14.5%) men with prevalent cancer at recruitment, defined as in Christakoudi et al.,^8^ missing anthropometric measurements, mismatch between the genetic and self‐reported sex, sex steroid treatment or prostate surgery (see Table S1 for details on exclusions and Table S2 for lists of excluded medications).

### 2.2 Prostate cancer ascertainment

Cancer cases in UK Biobank are ascertained based on linkage to the national cancer registry of the United Kingdom. The outcome of interest was total prostate cancer incidence, defined as the first primary prostate cancer diagnosed after recruitment, with code C61 from the 10th version of the International Statistical Classification of Diseases (ICD10) and malignant behaviour (behavioural code 3 or 5), with no separation by grade or aggressiveness at diagnosis, as this information is not available in UK Biobank. Follow‐up was censored at the date of diagnosis for first primary incident cancers in locations other than the prostate, defined as in Christakoudi et al.,^8^ excluding non‐melanoma skin cancers but including skin squamous‐cell carcinomas. Follow‐up was censored for all men remaining cancer‐free at the date of the last complete cancer registry (31 March 2020 for England and Scotland, 31 December 2016 for Wales), or at the date of death, if earlier.

### 2.3 Anthropometric indices and diabetes status

Anthropometric measurements were obtained by dedicated technicians according to established protocols.^16^ WC was measured at the natural indent or the umbilicus and HC at the widest point. We calculated ABSI with coefficients from the National Health and Nutrition Examination Survey (NHANES)^12^ but HI with coefficients derived for UK Biobank men,^11^ as HI calculated with coefficients from NHANES^13^ was correlated inversely with BMI in UK Biobank men^8^:

$$\text{ABSI}=1000\times \mathrm{WC}\left(m\right)\times \text{Weight}{\left(\mathrm{kg}\right)}^{-2/3}\times \text{Height}{\left(m\right)}^{5/6}$$

$$\mathrm{HI}=\mathrm{HC}\left(\mathrm{cm}\right)\times \text{Weight}{\left(\mathrm{kg}\right)}^{-2/5}\times \text{Height}{\left(\mathrm{cm}\right)}^{1/5}$$

$$\mathrm{BMI}=\text{Weight}\left(\mathrm{kg}\right)\times \text{Height}{\left(m\right)}^{-2}$$

Diabetes status at recruitment was based on self‐reported information about diabetes mellitus (without distinction between type 1 and type 2), insulin treatment, treatment with antidiabetic medications (listed in Table S2)^17^ or glycated haemoglobin HbA1c ≥48 mmol/mol (see further details in Appendix S1).

### 2.4 Biomarker measurements

Blood samples were obtained throughout the day with no requirement for fasting. Biomarker measurements were performed by UK Biobank. Serum levels of oestradiol, testosterone and SHBG were measured with chemiluminescent immunoassays (competitive binding for sex steroids, two‐step sandwich for SHBG) on Beckman Coulter DXI 800 analyser.^18^ We calculated FT with law‐of‐mass‐action equations,^19^ using measured albumin. We used HbA1c as biomarker of glucose metabolism, as this is not affected by fasting and provides information for glucose status over the past 3 months. HbA1c was measured in red blood cells with high‐performance liquid chromatography on Bio‐Rad VARIANT II Turbo analyser. To accommodate undetected values for oestradiol in men (91%), we considered oestradiol dichotomised at the lowest detected level (175 pmol/L). For testosterone, SHBG and HbA1c, values below or above the limits of detection were few and were set to either half the lowest detected level or the upper limit value, correspondingly.

### 2.5 Definition and selection of covariates

In addition to age at recruitment, we selected the following potential confounders a priori, based on literature reports of their associations with the exposures and the outcome (see related references in Table S3): height, weight change within the year preceding recruitment (weight loss, stable weight, weight gain), smoking status (never, former occasional, former regular, current), alcohol consumption (≤3 times/month, ≤4 times/week, daily), physical activity (less active, moderately active, active), Townsend deprivation index quintiles (as a proxy of socio‐economic status) and family history of cancer (none, breast/lung/bowel, prostate). Given that we examined associations with biomarkers in blood, we also considered fasting time (0–2 h, 3–4 h, ≥5 h) and time of blood collection (<12:00, 12:00 to <16:00, ≥16:00) (see further details on definitions in Appendix S1). We then examined their pairwise associations with the exposures (using linear regression models for anthropometric indices, testosterone, and SHBG, and logistic regression models for oestradiol) and with the outcome, prostate cancer risk (using Cox proportional hazards models) (Figure S1). We excluded physical activity and fasting time from the final list of covariates, as these were not associated with prostate cancer risk. We retained the remaining covariates because these were associated with at least some of the exposures, as well as with prostate cancer risk. We used only family history of prostate cancer as family history of other cancers was not associated with prostate cancer risk.

### 2.6 Statistical analysis

We used Stata‐13 for the statistical analyses and R version 4.1.3^20^ for data management.

In the main analyses, we used BMI, ABSI and HI on a standardised continuous scale (*z*‐scores, value minus mean, divided by standard deviation, SD). We estimated hazard ratios (HR) and 95% confidence intervals (CI) with delayed‐entry Cox proportional hazards models, which are conditional on surviving free of cancer to cohort recruitment. The underlying time scale was age, with origin at the date of birth, entry time was the date at recruitment, and exit time was the date of diagnosis of the first incident cancer, or death, or last complete follow‐up, whichever occurred first. We first examined a model including BMI, ABSI, HI (per one SD increment, HR_per_SD_) and diabetes (yes vs. no, HR_Yes_No_) as exposures. We then examined models additionally including either FT and SHBG jointly as exposures, or oestradiol or TT individually. All models were stratified by age at recruitment, region of the assessment centre, and family history of prostate cancer, and adjusted for height (z‐scores), recent weight change, smoking status, alcohol consumption, Townsend deprivation index and time of sample collection. We tested the proportional hazards assumption based on Schoenfeld residuals and established that family history of prostate cancer must be used as a stratifying variable for the assumption to be valid. Missingness for covariates and diabetes status was very low (<1% for all, except <2% for recent weight change). Nevertheless, we performed multiple sequential imputations with chained equations (function *mi impute* in Stata 13, *m* = 5 imputed datasets) using multinomial logistic regression models (for recent weight change, smoking status, alcohol consumption, Townsend deprivation index quintiles and time of sample collection) or a logistic regression model (for diabetes status), with stratification for region and adjustment for age at recruitment, BMI, ABSI and family history of prostate cancer. We derived the estimates of coefficients and standard errors with Rubin's combination rules (function *mi estimate* in Stata‐13).^21^ Tests of statistical significance were two‐sided.

To explore heterogeneity by BMI and diabetes status, we examined groups of men according to World Health Organisation categories of BMI (normal weight BMI <25 kg/m^2^; overweight BMI = 25 to <30 kg/m^2^; obese BMI ≥30 kg/m^2^) and groups of men with and without diabetes. Only for subgroup analysis, we considered men with unknown diabetes status as no‐diabetes because group size was not allowed to vary between the imputed datasets. We evaluated multiplicative interactions with BMI on a continuous scale or with diabetes status with Wald test for the corresponding interaction term, included for each anthropometric index or biomarker individually in the fully adjusted model (*p*_MI_). We additionally calculated the Relative Excess Risk from Interaction with obesity or diabetes status (RERI; ranges between – infinity and + infinity; null value 0), the attributable proportion due to interaction (AP; ranges between −1 and + 1; null value 0), and the ratio between combined and individual effects (synergy index (SI); ranges between 0 and + infinity; null value 1)^22^ from fully adjusted models including a cross‐classification of each body shape index or biomarker individually (dichotomised as high/low with respect to ≥median, or ≥175 pmol/L for oestradiol detection) with either obese (BMI ≥30 kg/m^2^, yes/no) or diabetes (yes/no), or a cross‐classification between obese and diabetes. To ensure that all three measures of interaction on the additive scale are directionally consistent with each other, we used as reference the cross‐classification category with the lowest observed prostate cancer risk, as recommended in.^23^ Thus, in the equations below, HR_High‐High_ indicates the cross‐classification category expected to have the highest prostate cancer risk when the two examined factors were coded as the opposite states to those in the reference cross‐classification category with the observed lowest risk. This approach defined RERI, AP and SI with respect to excess risk:

$$\text{RERI}={\mathrm{HR}}_{\text{High}-\text{High}}-{\mathrm{HR}}_{\text{High}-\mathrm{Low}}-{\mathrm{HR}}_{\mathrm{Low}-\text{High}}+1$$

$$\mathrm{AP}=\frac{\text{RERI}}{{\mathrm{HR}}_{\text{High}-\text{High}}}$$

$$\mathrm{SI}=\frac{\left({\mathrm{HR}}_{\text{High}-\text{High}}-1\right)}{\left({\mathrm{HR}}_{\text{High}-\mathrm{Low}}-1\right)+\left({\mathrm{HR}}_{\mathrm{Low}-\text{High}}-1\right)}$$

We calculated confidence intervals and *p*‐values (*p*_RERI_, *p*_AR_, *p*_SI_) with the multiple imputations equivalent of the delta method for non‐linear combinations (*mi estimate* in Stata‐13).^21^ For SI, we calculated confidence intervals for log‐transformed SI and transformed these back to the SI scale, similarly to the approach used in function *reri*, which we could not use because it is only available in the latest release of Stata.^24^

To explore potential non‐linearity, we examined quintile categories of ABSI, HI, FT, TT and SHBG, decile categories of FT and TT, and detailed categories of BMI according to,^25^ and tested non‐linearity with the Wald test for the non‐linear (second spline) term from fully adjusted models including restricted cubic splines for the exposure of interest (knots at ±2SD and the mean).

For comparison with traditional body shape measures, we examined associations of WC and HC individually with total prostate cancer risk, adjusting for height, diabetes status and covariates.

### 2.7 Sensitivity analyses

To explore the influence of covariates, we compared a minimally adjusted model (including BMI, ABSI, HI and height, and stratified by age at recruitment) with a model additionally stratified by region and family history of prostate cancer risk and further adjusted for covariates, and with the model additionally including diabetes status (the main fully adjusted model) and calculated adjustment differences in HR estimates for BMI, ABSI and HI compared to the minimally adjusted model. To examine the influence of adjustment for biomarkers, we calculated adjustment differences in HR estimates for BMI, ABSI, HI and diabetes status, comparing fully adjusted models with and without biomarkers, restricted to men with available biomarker measurements, and for HbA1c, also to men without known diabetes. To explore reverse causality, we excluded from the fully adjusted model men with less than 2 years of follow‐up, lagged the entry time with 2 years, and calculated lag differences in HR estimates for BMI, ABSI, HI and diabetes status compared to the fully adjusted model including all men. We have highlighted adjustment or lag differences ≥2%.

## 3 RESULTS

### 3.1 Cohort characteristics

During a mean follow‐up of 10.3 years, 9417 incident prostate cancers were ascertained in 195,813 men with mean BMI = 27.8 kg/m^2^ and mean ABSI = 79.8 (Table 1). Less than 1% of men (*n* = 442) were underweight (BMI <18.5 kg/m^2^), so we included these in the normal weight category. Half of men were overweight, one quarter were obese, and 13,987 (7.1%) had diabetes. There were large differences in WC and HC between normal weight and obese men, but differences in ABSI and HI were minimal. Men with diabetes had higher BMI and larger ABSI compared to men without diabetes, with little difference in HI. Obese men were more likely to have diabetes compared to normal weight men. TT, FT and SHBG were lower in obese men and men with diabetes compared to normal weight men and men without diabetes, correspondingly, while oestradiol was higher in obese men, with minimal difference according to diabetes status (Table 1). Obese men and men with diabetes were older at recruitment, had higher Townsend deprivation index and were more likely to be former regular smokers but less likely to consume alcohol or to have family history of prostate cancer compared to, correspondingly, non‐obese men and men without diabetes (Table S4). Obese men, however, were more likely to have gained weight during the year preceding recruitment compared to non‐obese men, while men with diabetes were more likely to have lost weight compared to men without diabetes (Table S4).

**TABLE 1:** Anthropometric characteristics and biomarker levels by BMI and diabetes category.

Groups	Overall	Normal weight	Overweight	Obese	Diabetes No	Diabetes Yes
Cohort: *n* (%)	195,813	49,175 (25.1)	96,775 (49.4)	49,863 (25.5)	181,826 (92.9)	13,987 (7.1)
Cases: *n* (%)	9417	2379 (25.3)	4938 (52.4)	2100 (22.3)	8825 (93.7)	592 (6.3)
Rate: per 1 × 10^6^ p.y.	4673	4675	4950	4127	4699	4316
Follow‐up (years)	10.3 (2.4)	10.3 (2.3)	10.3 (2.3)	10.2 (2.4)	10.3 (2.3)	9.8 (2.8)
Age at baseline	57.0 (8.1)	56.5 (8.3)	57.2 (8.1)	57.2 (7.9)	56.8 (8.1)	60.3 (6.9)
Anthropometry^a^						
Height (cm)	175.9 (6.8)	176.5 (6.8)	175.9 (6.7)	175.3 (6.8)	176.0 (6.8)	174.7 (6.8)
Weight (kg)	86.2 (14.3)	72.3 (7.2)	84.7 (7.7)	102.7 (12.9)	85.5 (13.8)	95.5 (17.6)
BMI (kg/m^2^)	27.8 (4.2)	23.2 (1.5)	27.4 (1.4)	33.4 (3.4)	27.6 (4.0)	31.2 (5.3)
ABSI	79.8 (4.1)	79.5 (4.4)	79.7 (4.0)	80.2 (4.0)	79.6 (4.1)	81.3 (4.1)
HI	49.1 (1.7)	49.3 (1.6)	49.0 (1.6)	49.2 (2.0)	49.1 (1.7)	49.1 (2.1)
WC (cm)	96.9 (11.3)	85.7 (6.2)	95.9 (6.2)	110.0 (9.6)	96.2 (10.8)	106.3 (13.3)
HC (cm)	103.5 (7.6)	96.9 (4.6)	102.7 (4.5)	111.5 (7.6)	103.2 (7.2)	107.9 (9.9)
Diabetes: *n* (%)						
Yes	13,987 (7.1)	1285 (2.6)	5104 (5.3)	7598 (15.2)	–	13,987 (100)
Biomarkers^b^						
E2: *n* (%)	15,438 (9.0)	3571 (8.3)	7317 (8.6)	4550 (10.4)	14,242 (8.9)	1196 (9.7)
TT (nmol/L)	11.4 (6.2–21.3)	12.9 (7.2–23.0)	11.5 (6.4–20.6)	10.1 (5.3–19.0)	11.6 (6.3–21.3)	9.8 (4.9–19.4)
FT (pmol/L)	240 (140–410)	250 (147–423)	243 (145–409)	224 (128–392)	242 (142–411)	213 (120–379)
SHBG (nmol/L)	36.5 (16.1–82.6)	44.2 (21.0–93.1)	35.8 (16.5–77.8)	31.3 (13.8–71.3)	36.8 (16.5–82.4)	32.3 (12.8–81.3)
HbA1c (mmol/mol)	35.7 (25.8–49.3)	34.4 (26.4–44.8)	35.2 (26.3–47.2)	37.8 (25.5–56.1)	34.6 (27.7–43.3)	51.9 (32.1–83.9)

*Note*: Comparisons between BMI categories and between diabetes status groups were performed with one‐way ANOVA for anthropometric indices and log‐transformed biomarkers and chi‐square test for categorical variables and oestradiol detection. All differences were significant at *p* < 0.0001, except *p* = 0.016 for HI and *p* = 0.003 for oestradiol detection comparing diabetes yes versus no.

Abbreviations: ABSI, a body shape index; BMI, body mass index; Cases, number of incident prostate cancer cases (percentage from total overall); Rate, number of incident prostate cancer cases per 1,000,000 person years of follow‐up in each group; E2, oestradiol (detected ≥175 pmol/L); FT, free testosterone; HbA1c, haemoglobin A1c (glycated haemoglobin); HC, hip circumference; HI, hip index; *n* (%), number of participants (percentage from total overall for cohort and cases or total per column for diabetes); Normal weight, BMI <25 kg/m^2^; Overweight, BMI ≥25 to <30 kg/m^2^; Obese, BMI ≥30 kg/m^2^; SD, standard deviation; SHBG, sex hormone binding globulin; TT, total testosterone; WC, waist circumference.

^a^ Mean (SD).

^b^ Geometric mean (reference range) in men with available E2 measurements (*n* = 171,858), or with available FT, TT and SHBG measurements (*n* = 168,091), or with available HbA1c measurements (*n* = 183,795).

### 3.2 Associations of diabetes and anthropometric indices with prostate cancer risk

**FIGURE 1:**
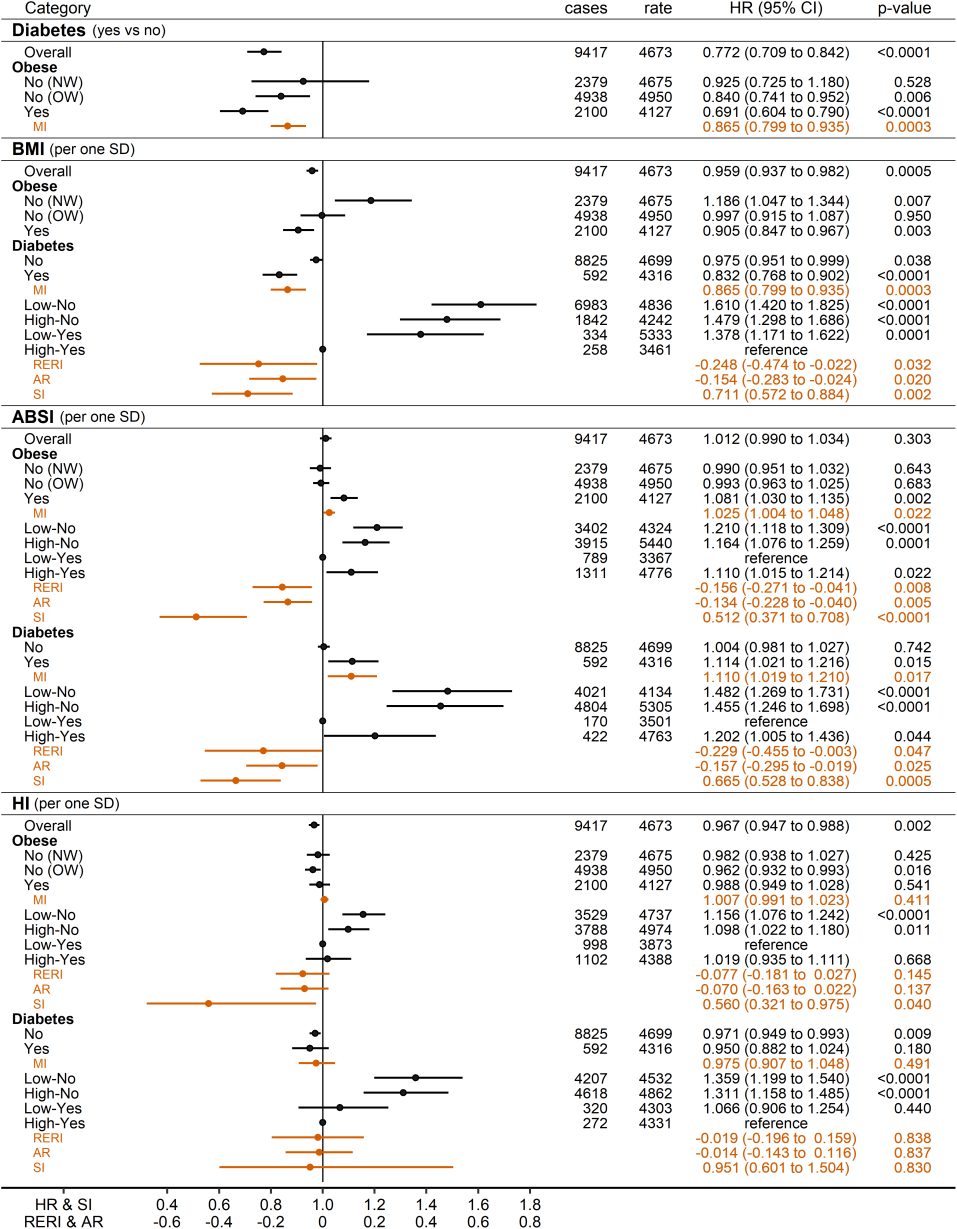
Associations of diabetes and anthropometric indices with prostate cancer risk. ABSI, a body shape index; AP, attributable proportion due to interaction; BMI, body mass index; cases, number of incident prostate cancer cases per group; CI, confidence interval; HI, hip index; HR, hazard ratio; MI, multiplicative interaction term for each of ABSI or HI individually (z‐scores) with either BMI (z‐scores) or diabetes (no/yes), or between BMI (z‐scores) and diabetes; NW, BMI <25 kg/m^2^; OW, BMI ≥25 to <30 kg/m^2^; Obese, BMI ≥30 kg/m^2^; rate, number of incident prostate cancer cases per 1,000,000 person years of follow‐up in each group; RERI, relative excess risk from interaction; SD, standard deviation; SI, synergy index. Estimates from Cox proportional hazards models in men overall, including diabetes (no/yes) and BMI, ABSI and HI (z‐scores, value minus mean divided by SD) as exposures, stratified by age at recruitment, region of the assessment centre and family history of prostate cancer, and adjusted for height, recent weight change, smoking status, alcohol consumption, Townsend deprivation index and time of blood collection. Low/High–No/Yes—groups of men according to a cross‐classification of each body shape index individually (low/high), dichotomised at the median (ABSI ≥79.8 or HI ≥49.1), with either obese or diabetes (no/yes), or a cross‐classification between obese and diabetes.

In men overall, diabetes was associated inversely with prostate cancer risk (HR_Yes_No_ = 0.772, 95% CI = 0.709–0.842), more strongly in obese men (HR_Yes_No_ = 0.691, 95% CI = 0.604–0.790) (Figure 1). Although BMI was also associated inversely in men overall (HR_per_SD_ = 0.959; 95% CI = 0.937 to 0.982) and more strongly in obese men (HR_per_SD_ = 0.905; 95% CI = 0.847–0.967), it was associated positively in normal weight men (HR_per_SD_ = 1.186, 95% CI = 1.047–1.344), with no evidence for association in overweight men. The inverse association with BMI was stronger in men with diabetes (HR_per_SD_ = 0.832, 95% CI = 0.768–0.902) than in men without diabetes (*p*_MI_ = 0.0003 for an inverse multiplicative interaction between BMI (z‐scores) and diabetes). Prostate cancer risk was lowest when obese and diabetes occurred together and the risk for non‐obese without diabetes (expected to be the highest‐risk group) was lower than the additive individual effects of non‐obese and no‐diabetes (RERI_NonObese_NoDiabetes_ = −0.248; 95% CI = −0.474 to −0.022; *p*_RERI_ = 0.032; *p*_AR_ = 0.020; *p*_SI_ = 0.002) (Figure 1), such that the inverse association was strongest when obesity and diabetes occurred together.

ABSI was associated positively with prostate cancer risk but only in obese men (HR_per_SD_ = 1.081, 95% CI = 1.030 to 1.135, *p*_MI_ = 0.022 for a positive multiplicative interaction with BMI) and in men with diabetes (HR_per_SD_ = 1.114, 95% CI = 1.021 to 1.216, *p*_MI_ = 0.017 for a positive multiplicative interaction with diabetes) (Figure 1). Prostate cancer risk was lowest when low‐ABSI <79.8 and obese occurred together and the risk for high‐ABSI ≥79.8 and non‐obese (expected to be the highest‐risk group) was lower than the additive individual effects of high‐ABSI and non‐obese (RERI_HighABSI_NonObese_ = −0.156; 95% CI = −0.271 to −0.041; *p*_RERI_ = 0.008; *p*_AR_ = 0.005; *p*_SI_ <0.0001). The association and interaction patterns were similar when considering diabetes instead of obesity (RERI_HighABSI_NoDiabetes_ = −0.229; 95% CI = −0.455 to −0.003; *p*_RERI_ = 0.047; *p*_AP_ = 0.025; *p*_SI_ = 0.0005), such that the inverse associations with both obesity and diabetes were attenuated for high‐ABSI. HI was associated inversely with prostate cancer risk in men overall (HR_per_SD_ = 0.967; 95% CI = 0.947–0.988), without strong evidence for interactions with BMI or diabetes status (Figure 1).

### 3.3 Associations of sex steroids with prostate cancer risk

**FIGURE 2:**
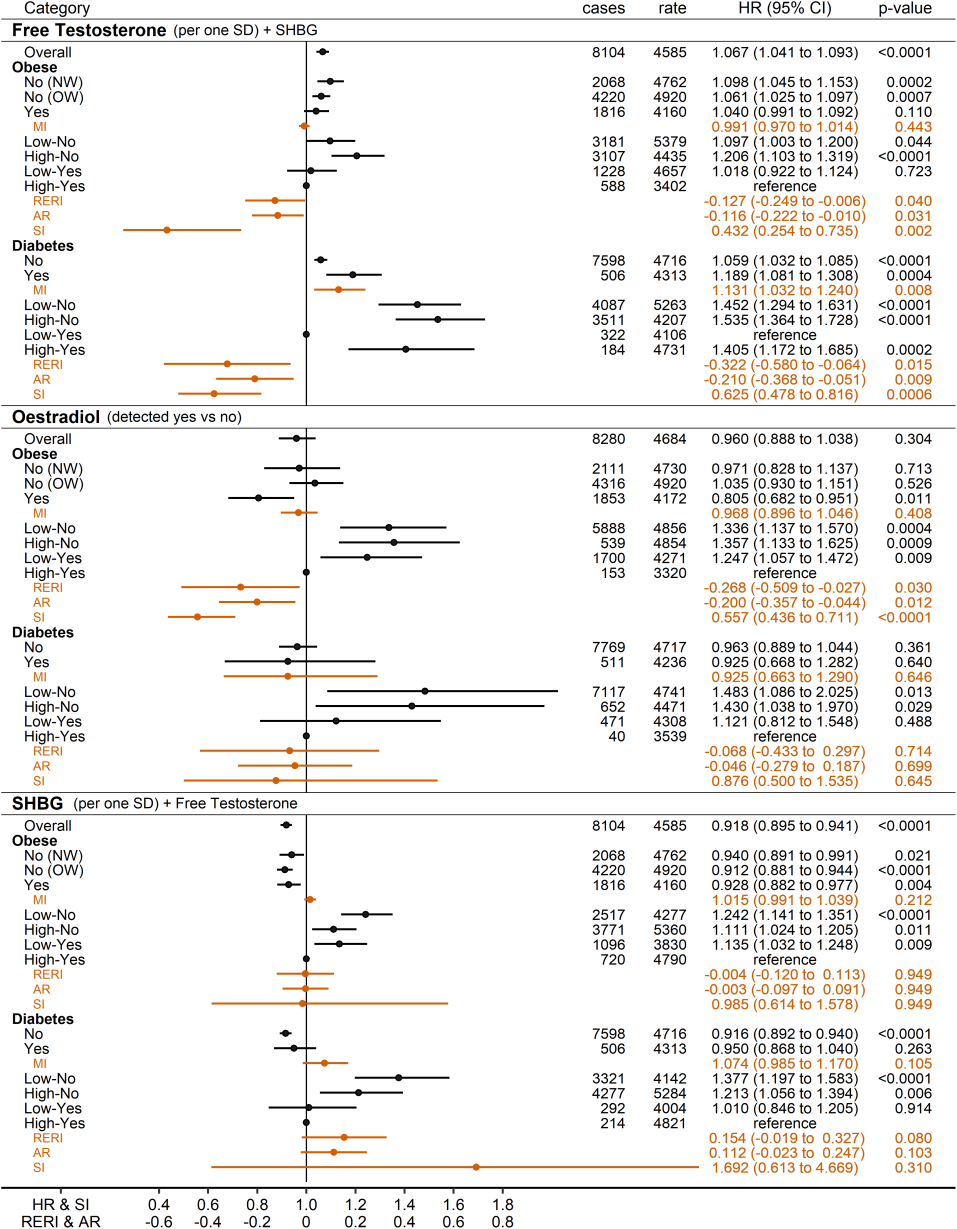
Associations of circulating sex steroids and SHBG with prostate cancer risk. AP, attributable proportion due to interaction; cases, number of incident prostate cancer cases per group; CI, confidence interval; HR, hazard ratio; MI, multiplicative interaction term for each biomarker individually (z‐scores for free testosterone and SHBG or oestradiol ≥175 pmol/L no/yes) with either BMI (z‐scores) or diabetes (no/yes); NW, BMI <25 kg/m^2^; OW, BMI ≥25 to <30 kg/m^2^; Obese, BMI ≥30 kg/m^2^; rate, number of incident prostate cancer cases per 1,000,000 person years of follow‐up in each group; RERI, relative excess risk from interaction; SD, standard deviation; SHBG, sex hormone binding globulin; SI, synergy index. Estimates from Cox proportional hazards models in men overall with available total and free testosterone and SHBG measurements (*n* = 168,091) or with available oestradiol measurement (*n* = 171,858), including either jointly free testosterone and SHBG (z‐scores, value minus mean divided by SD, following log‐transformation), or individually oestradiol (≥175 pmol/L yes/no), stratified by age at recruitment, region of the assessment centre and family history of prostate cancer, and adjusted for diabetes status, BMI, ABSI, HI, and height (z‐scores), recent weight change, smoking status, alcohol consumption, Townsend deprivation index and time of blood collection. Low/High–No/Yes—groups of men according to a cross‐classification of each biomarker individually (low/high), dichotomised at the median (free testosterone ≥243 pmol/L; SHBG≥37.1 nmol/L) or at the lowest detected level for oestradiol (≥175 pmol/L), with either obese or diabetes (no/yes).Associations and interactions with total testosterone and multiplicative interactions of sex steroids and SHBG with body shape indices are shown in Figure S2.

FT was associated positively with prostate cancer risk in men overall (HR_per_SD_ = 1.067; 95% CI = 1.041 to 1.093), but most strongly in normal weight men (HR_per_SD_ = 1.098; 95% CI = 1.045 to 1.153), with less evidence for association in obese men, although without evidence for a multiplicative interaction with BMI (*p*_MI_ = 0.443, Figure 2). Prostate cancer risk, however, was lowest when high‐FT ≥243 pmol/L and obese occurred together and the risk for low‐FT and non‐obese (expected to be the highest‐risk category) was lower than the additive individual effects of low‐FT and non‐obese (RERI_LowFT_NonObese_ = −0.127; 95% CI = −0.249 to −0.006; *p*_RERI_ = 0.040; *p*_AR_ = 0.031; *p*_SI_ = 0.002), such that the inverse association with obese was stronger for high‐FT. The positive association with FT was stronger in men with diabetes (HR_per_SD_ = 1.189; 95% CI = 1.081 to 1.308) than in men without diabetes (*p*_MI_ = 0.008 for a positive multiplicative interaction with diabetes). Prostate cancer risk was lowest when low‐FT and diabetes occurred together and the risk for high‐FT without diabetes (expected to be the highest‐risk category) was lower than the additive individual effects of high‐FT and no‐diabetes (RERI_HighFT_NoDiabetes_ = −0.322; 95% CI = −0.580 to −0.064; *p*_RERI_ = 0.015; *p*_AR_ = 0.009; *p*_SI_ = 0.0006), such that the inverse association with diabetes was attenuated for high‐FT (Figure 2). TT was similarly associated positively with prostate cancer risk only in normal weight men and in men with diabetes, although there was less evidence for interactions with obese or diabetes (Figure S2).

Oestradiol was associated inversely with prostate cancer risk only in obese men (HR_Yes_No_ = 0.805; 95% CI = 0.682 to 0.951 for oestradiol ≥175 pmol/L), although without evidence for a multiplicative interaction with BMI (*p*_MI_ = 0.408) (Figure 2). Prostate cancer risk was lowest when high‐oestradiol and obese occurred together, and the risk for low‐oestradiol and non‐obese (expected to be the highest‐risk category) was lower than the additive individual effects of low‐oestradiol and non‐obese (RERI_LowOestradiol_NonObese_ = −0.268; 95% CI = −0.509 to −0.027; *p*_RERI_ = 0.030; *p*_AP_ = 0.012; *p*_SI_ <0.0001), such that the inverse association with obesity was stronger for high‐oestradiol. There was no evidence for interaction of oestradiol with diabetes (Figure 2).

Further adjustment of models for oestradiol for FT and SHBG and adjustment of models for FT for oestradiol made no material difference to the estimates (Figure S3).

SHBG was associated inversely with prostate cancer risk in men overall (HR_per_SD_ = 0.918; 95% CI = 0.895 to 0.941), with little evidence for interactions with obesity or diabetes (Figure 2), but with some evidence for an inverse multiplicative interaction with HI (*p*_MI_ = 0.024) (Figure S2).

### 3.4 Non‐linearity of the associations with prostate cancer risk

**FIGURE 3:**
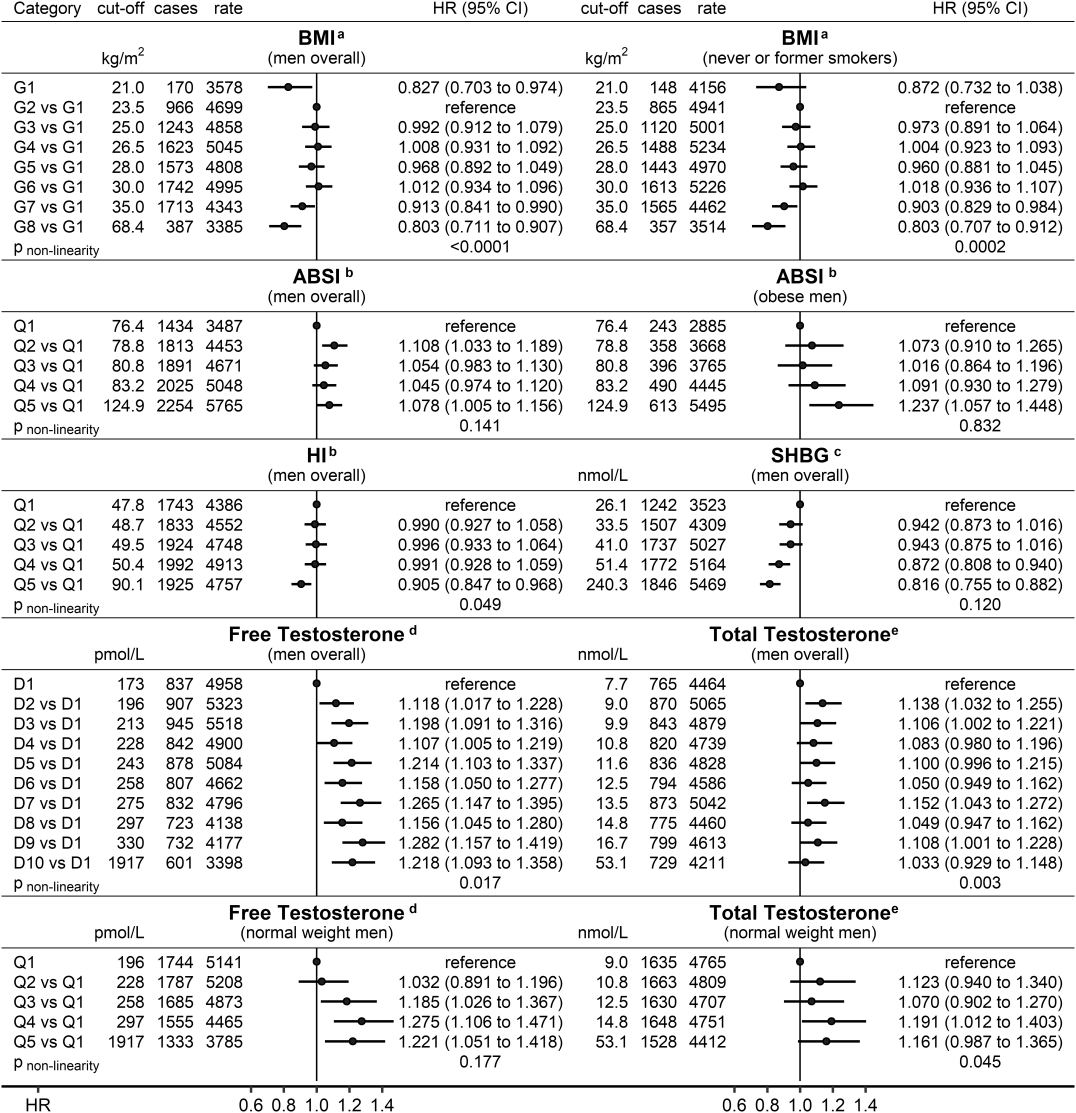
Associations of anthropometric index and biomarker categories with prostate cancer risk. ABSI, a body shape index; BMI, body mass index; cases, number of incident prostate cancer cases per group; CI, confidence interval; cut‐off, upper boundary of the group; D1‐D10, deciles; G1‐G8, BMI categories according to^25^; HI, hip index; HR, hazard ratio; Q1‐Q5, quintiles; rate, number of incident prostate cancer cases per 1,000,000 person years of follow‐up in each group; SD, standard deviation; SHBG, sex hormone binding globulin. ^a^Cox proportional hazards models, including BMI categories, ABSI and HI (z‐scores, value minus mean divided by SD), stratified by age at recruitment, region of the assessment centre and family history of prostate cancer, and adjusted for diabetes status, height, recent weight change, smoking status, alcohol consumption, Townsend deprivation index and time of blood collection in men overall (*n* = 195,813, left) or excluding current smokers (*n* = 171,525, right); ^b^Like ‘a’ but including quintiles either for ABSI or HI and z‐scores for BMI in men overall and, for ABSI, additionally restricted to obese men (BMI ≥30 kg/m^2^; *n* = 49,863, right); ^c^Like ‘a’, but in men overall with available total and free testosterone and SHBG measurements (*n* = 168,091), including as exposure SHBG quintiles adjusted for free testosterone (z‐scores, following log‐transformation) and z‐scores for BMI; ^d^Like ‘c’, but including as exposure free testosterone deciles in men overall (*n* = 168,091), or free testosterone quintiles in normal weight men (BMI <25 kg/m^2^; *n* = 42,011), with adjustment for SHBG (z‐scores, following log‐transformation); ^e^Like ‘d’, but including as exposure total testosterone deciles (men overall) or quintiles (normal weight men) without adjustment for SHBG; *p*_non‐linearity_—*p*‐value from Wald test for the non‐linear (second spline) term from fully adjusted models including restricted cubic splines for the exposure of interest (knots at ±2SD and the mean).

There was strong evidence for non‐linearity of the association of BMI with prostate cancer risk in men overall (*p*_non‐linearity_ <0.0001), with similar risk for BMI between 21.0 and 30.0 kg/m^2^ and lower risk for obese BMI (Figure 3). Although the risk was also lower for very low BMI (<21 kg/m^2^), this association was partly attenuated after removing current smokers. There was no evidence for non‐linearity of the positive association with ABSI in obese men. In men overall, prostate cancer risk was similarly higher for all ABSI quintiles compared to the lowest but was lower only for the highest HI quintile (*p*_non‐linearity_ = 0.049). There was little evidence for non‐linearity of the inverse association with SHBG. The positive associations with FT and TT plateaued at high levels in men overall, with higher risk for all deciles compared to the lowest (*p*_non‐linearity_ = 0.017 for FT; *p*_non‐linearity_ = 0.003 for TT), but with less evidence for non‐linearity in normal weight men (Figure 3).

### 3.5 Comparisons with waist and hip circumferences

**FIGURE 4:**
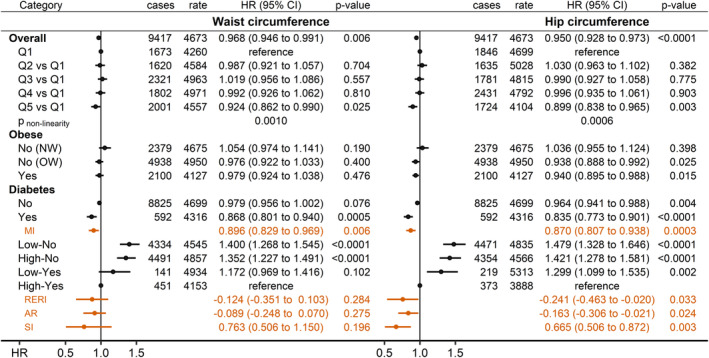
Associations of waist and hip circumferences with prostate cancer risk. AP, attributable proportion due to interaction; cases, number of incident prostate cancer cases per group; CI, confidence interval; HR, hazard ratio; MI, multiplicative interaction term for either waist or hip circumference (z‐scores) with diabetes (no/yes); NW, BMI <25 kg/m^2^; OW, BMI ≥25 to <30 kg/m^2^; Obese, BMI ≥30 kg/m^2^; Q1‐Q5, quintiles (cut‐offs: 88, 93, 99, 105 cm for waist circumference; 98, 101, 104, 109 cm for hip circumference); rate, number of incident prostate cancer cases per 1,000,000 person years of follow‐up in each group; RERI, relative excess risk from interaction; SD, standard deviation; SI, synergy index. Estimates from Cox proportional hazards models, including individually waist or hip circumference (z‐scores, value minus mean divided by SD) as exposure, stratified by age at recruitment, region of the assessment centre and family history of prostate cancer, and adjusted for diabetes status, height, recent weight change, smoking status, alcohol consumption, Townsend deprivation index and time of blood collection. Low/High–No/Yes—groups of men according to a cross‐classification of either waist or hip circumference (low/high, dichotomised at the median: waist circumference ≥96 cm; hip circumference ≥103 cm) with diabetes (no/yes). *p*_non‐linearity_—*p*‐value from Wald test for the non‐linear (second spline) term from fully adjusted models including restricted cubic splines either for waist or hip circumference (knots at ±2SD and the mean).

Both WC and HC showed inverse associations with prostate cancer risk, although weaker for WC than for HC (Figure 4). For both WC and HC, prostate cancer risk was lower specifically for the highest compared to the lowest quintile (*p*_non‐linearity_ = 0.001 for WC; *p*_non‐linearity_ = 0.0006 for HC). Also, for both WC and HC, the inverse association was stronger in men with diabetes than in men without diabetes, with evidence for an inverse multiplicative interaction with diabetes (*p*_MI_ = 0.006 for WC, *p*_MI_ = 0.0003 for HC), and with the lowest prostate cancer risk when high‐WC ≥96 cm or high‐HC≥103 cm occurred together with diabetes. Only for HC, however, there was consistent evidence for an additive interaction with diabetes, with a lower risk for low‐HC without diabetes (expected to be the highest‐risk category) than the additive individual effects of low‐HC and no‐diabetes (RERI_LowHC_NoDiabetes_ = −0.241; 95% CI = −0.463 to −0.020; *p*_RERI_ = 0.033; *p*_AP_ = 0.024; *p*_SI_ = 0.003) (Figure 4).

### 3.6 Sensitivity analyses

The associations of BMI with prostate cancer risk were attenuated in the fully adjusted compared to the minimally adjusted models, with mainly the adjustment for covariates attenuating the positive association in normal weight men but mainly the adjustment for diabetes status attenuating the inverse association in obese men (Table S5). Restricting the analysis to men with at least 2 years of follow‐up had little influence on association estimates. Adding sex steroids and SHBG to the fully adjusted main models had little influence, except for some attenuation of the inverse association with BMI in men with diabetes after adjustment for TT. In men without diabetes, adjustment for HbA1c had no material influence on association estimates and there was little evidence for association of HbA1c with prostate cancer risk (HR_per_SD_ = 0.983; 95% CI = 0.961 to 1.006 in men overall; HR_per_SD_ = 0.974; 95% CI = 0.928 to 1.022 in obese men), even for pre‐diabetic levels (HR = 0.968; 95% CI = 0.864 to 1.085 for HbA1c ≥42 to <48 compared to HbA1c <42 mmol/mol) (Table S5).

## 4 DISCUSSION

In this study, obesity and diabetes were associated more strongly with lower total prostate cancer risk when occurring together. For high ABSI, total prostate cancer risk was higher in obese men and men with diabetes and the inverse associations with obesity and diabetes were attenuated. For high circulating FT, total prostate cancer risk was higher in non‐obese men and men with diabetes and the inverse association with obesity was stronger but the inverse association with diabetes was attenuated. Associations with TT were similar but weaker. For high oestradiol, prostate cancer risk was lower in obese men and the inverse association with obesity was stronger. HI and SHBG were associated inversely with prostate cancer risk, more strongly when both were high.

A recent umbrella meta‐analysis concluded that the evidence supporting an inverse association of BMI with prostate cancer risk was suggestive for low‐grade (4 studies) and weak for non‐advanced prostate cancer (14 studies) but there was a null association for total prostate cancer risk.^4^ A further meta‐analysis, including 17 prospective studies, similarly reported an inverse association for nonaggressive prostate cancer but with significant heterogeneity.^26^ The main outlier reporting a positive association, however, was a cohort study in high‐risk men undergoing a baseline prostate biopsy on suspicion of prostate cancer.^27^ Although we could not discriminate prostate cancers by stage or grade in our study, our findings are in agreement with a study in the European Prospective Investigation into Cancer and Nutrition (EPIC) cohort, reporting inverse associations of BMI with total prostate cancer, as well as with localised and low‐intermediate grade,^28^ suggesting that the larger part of prostate cancers in UK Biobank men are localised and low‐grade. Our findings of an inverse association with BMI only in obese men, with strong evidence for non‐linearity, differ from a dose–response meta‐analysis of 12 earlier prospective studies, which found no evidence of non‐linearity for localised prostate cancer.^29^ Our findings, however, are consistent with more recent large studies in European men reporting inverse associations with total and low‐intermediate grade prostate cancer for BMI above 27 kg/m^2^,^28^, ^30^ and with a previous UK Biobank study reporting lower risk for obese but not for overweight compared to normal weight men.^9^ Furthermore, a large study in Swedish men reported lower risk of total and low‐intermediate grade prostate cancer for BMI <22.5 kg/m^2^,^30^ in agreement with the lower risk for BMI <21 kg/m^2^ in our study. The latter association, however, was partly attenuated in our study after removing current smokers and may thus reflect some influence of smoking, which is associated inversely with prostate cancer risk.^31^ Regarding the inverse association with diabetes, a meta‐analysis including 45 studies (29 cohort, 16 case–control) concluded that this was supported by strong evidence, with no difference between cohort and case–control studies.^3^ Consistent with our findings, lower prostate cancer risk has previously been reported for UK Biobank men only for high HbA1c (at least 42 mmol/mol),^32^, ^33^ suggesting that sustained hyperglycaemia is required. A novel contribution of our study is showing that obesity and diabetes facilitate each other for their inverse associations with prostate cancer risk.

Our findings of a null association of ABSI with prostate cancer risk in men overall are consistent with a previous report for UK Biobank including all ethnicities,^10^ but differ from an inverse association reported for the Australian and New Zealand Diabetes and Cancer Collaboration of prospective studies.^34^ The inverse association with ABSI in the latter report, however, was prominent only for ever smokers, comprising over 60% of men compared to 40% for ever regular smokers in UK Biobank, and could thus reflect the influence of smoking, which is associated with lower prostate cancer risk^31^ and higher visceral adiposity.^35^, ^36^ A larger number of previous studies have examined associations with WC, with a null association reported by an umbrella meta‐analysis for total prostate cancer risk and for non‐advanced stage and a positive association only for advanced stage.^4^ In UK Biobank and EPIC, however, the association pattern of WC with total and low‐intermediate grade prostate cancer resembled BMI, with an inverse association more specifically for high WC, consistent with the findings of our study, and a positive association only for high‐grade prostate cancer,^9^, ^28^, ^33^ corresponding to the strong positive correlation between BMI and WC. Thus, when the influence of factors related to fat distribution differs from the influence of factors related to fat accumulation with respect to the outcome of interest, WC provides similar information to BMI, while ABSI can reflect the differences because it is not correlated with BMI. To our knowledge, associations of HI with prostate cancer risk have not been examined by other authors. The suggestive inverse association from our earlier study in UK Biobank^8^ was statistically significant in this study, which included a larger number of cases. Few studies have also examined associations with HC, but findings in EPIC were similar to our study, with the association pattern of HC resembling BMI and WC,^28^ corresponding to their strong positive correlations.

Our findings are further in agreement with a previous study of prostate cancer risk in UK Biobank, reporting positive associations with FT and inverse with SHBG based on quintile categories and fewer cases.^37^ They are also in agreement with a recent pooled analysis of 25 nested case–control studies and a Mendelian Randomisation analysis by the Endogenous Hormones, Nutritional Biomarkers and Prostate Cancer Collaborative Group.^38^ This showed positive associations of FT with total (15,000 cases), as well as with low grade, and nonaggressive prostate cancer, clearer in normal weight men and stronger in men with diabetes, although an inverse association with SHBG was noted only in the observational and not in the MR analysis and an inverse association with TT was noted in the observational analysis,^38^ likely influenced by the strong positive association of TT with SHBG, which we have previously described.^6^ FT has additionally been associated with higher risk of ERG‐positive prostate cancers, which have an androgen‐induced fusion between the androgen‐regulated TMPRSS2 gene and the oncogene ERG.^39^ The null association of oestradiol with prostate cancer risk in men overall reported in our study is consistent with previous smaller‐scale studies.^40^, ^41^ The inverse association in obese men, however, is unexpected because it is currently believed, based on animal studies and prostate cancer cell lines, that oestrogens cooperate with androgens to facilitate prostate cancer development and A‐ring hydroxylated oestrogens lead to DNA adducts, DNA oxidative damage and lipid peroxidation.^42^ Nevertheless, no clear positive association of circulating oestradiol with prostate cancer risk has been demonstrated in prospective studies to date and previous studies have not examined oestradiol specifically in obese men.

Our findings support an involvement of testosterone suppression in the inverse association with diabetes, as FT, which is considered the active and biologically relevant part of testosterone, was lower in men with diabetes and high FT attenuated the inverse association with diabetes, such that the risk was lower in men with diabetes only when FT was low. This disagrees with suggestions that lower prostate cancer risk in individuals with diabetes simply reflects delayed diagnosis^43^ and disagrees with smaller‐scale studies, which have found limited evidence for inverse associations of diabetes or hyperglycaemia with testosterone levels and have thus considered testosterone involvement unlikely.^44^, ^45^ In the considerably larger UK Biobank, however, our current study and a previous study show lower TT and FT levels for diabetes (based on self‐reported diagnosis and antidiabetic treatment) and for HbA1c ≥42 mmol/mol.^33^ Low testosterone has also been associated not only with higher insulin resistance in cross‐sectional studies but with higher risk of developing insulin resistance in prospective studies.^45^ Although testosterone treatment in men with low testosterone can reduce glycaemia,^46^ glycaemia improvements are not consistent across studies and routine testosterone testing and treatment is not recommended in men with type 2 diabetes without clinical symptoms of testosterone deficiency.^47^ Our study indicates that low testosterone is required for the inverse association of diabetes with prostate cancer risk, suggesting a mechanistic role of diabetes in risk reduction, at least for low‐grade prostate cancers.

The role of testosterone for the inverse association of obesity with prostate cancer risk appears more complicated. Although TT and FT are lower in obesity, high testosterone facilitated rather than attenuated the inverse association with obesity as did high oestradiol, suggesting that testosterone acts as substrate for adipose tissue aromatise and oestradiol generation. In accordance, TT and FT were correlated strongly positively with each other and both were correlated positively with oestradiol.^6^ It would be important, however, to clarify whether testosterone and oestradiol strengthen the inverse association with obesity specifically for low‐grade prostate cancer and whether their relationship with high‐grade prostate cancer is different. Notably, in a large, pooled analysis, FT was associated positively with aggressive prostate cancer only in men younger than 60 years at blood collection but inversely in older men.^38^ Furthermore, while androgen receptor positivity increases in advanced and metastatic prostate cancers, oestrogen receptor ERα expression peaks for high‐grade prostatic intraepithelial neoplasia and is reduced for metastatic prostate cancer.^48^ Differences in sex steroid metabolism in prostate tissues of obese and non‐obese men may also be relevant, as oestrogen receptor ERα and aromatase expression is lower in the stroma of non‐cancerous prostate acini from obese compared to non‐obese prostate cancer patients.^49^

The attenuation by ABSI of the inverse associations of obesity and diabetes with total prostate cancer risk would not involve testosterone, because high ABSI is associated with lower TT and FT^6^ and testosterone administration reduces ABSI in men.^50^ This could, however, reflect the positive association of waist size with high‐grade prostate cancers, which are more common among obese men,^1^, ^4^ highlighting the need to clarify whether ABSI is associated positively with low‐grade prostate cancers. The inverse association with HI is unlikely to involve oestradiol, because HI was associated positively with oestradiol mainly in obese men,^6^ while the inverse association of HI with total prostate cancer risk did not show heterogeneity between BMI categories. Furthermore, HI reflects differences in gluteofemoral fat mainly in obese men but, in non‐obese men, reflects differences in gluteofemoral lean mass.^11^ Similarly, the inverse association of SHBG with prostate cancer risk did not show heterogeneity according to BMI. It is unclear, therefore, what is the mechanism underlying the synergistic inverse associations of HI and SHBG with total prostate cancer risk.

A clear strength of our study is the prospective cohort design and the large sample size for incident prostate cancer cases and for biomarker measurements, which permitted examining subgroups and cross‐classifications. Anthropometry was performed by trained personnel according to standardised protocols, avoiding bias from self‐reporting. We chose ABSI and HI as the appropriate indices of body shape, since by design they effectively account for the high correlations of WC and HC with height, weight and BMI, which is not true for popular indices of central body obesity such as the waist‐to‐height ratio, body roundness index, conicity index, weight‐adjusted waist index among others, which retain corelations or even introduce stronger correlations with height or BMI.^51^ Unified quality control procedures were applied to biomarker measurements, minimising measurement errors. Information for major lifestyle factors was available, permitting adjustment and minimising confounding. Some information for oestradiol levels, albeit limited, was also available for most of the cohort.

A major limitation of our study is the low sensitivity of the oestradiol assay, which permitted identification only of the top tenth of the distribution. The dichotomisation led to loss of information and prevented calculation of free oestradiol. Like other observational studies, we did not have FT measurements and relied on law‐of‐mass‐action equations, which may not be valid for obesity or diabetes. Blood samples were collected with no requirement for fasting and throughout the day, which could have contributed to a larger variability, although we have adjusted for time of blood collection and found no association of fasting time with prostate cancer risk. We had information for exposures only at baseline and could not account for changes during follow‐up. Importantly, we did not have information for prostate cancer grade and stage. We could not separate type 1 from type 2 diabetes either, although hyperglycaemia‐related pathways would be relevant to both types. The definition of diabetes had to rely on self‐reported information, which may be incomplete, and on a single HbA1c measurement. We were unable to clarify ethnic differences, which would be important because sex steroid levels and associations of diabetes with prostate cancer risk differ between ethnic groups.^3^, ^52^ Last, UK Biobank is not representative of the general population and includes participants with healthier lifestyle.^53^

In conclusion, our study showed synergistic inverse associations of obesity and diabetes with total prostate cancer risk, attenuated in men with high‐ABSI and, for diabetes, additionally attenuated in men with high‐FT. The inverse association with obesity, however, was apparently facilitated by high‐oestradiol and by high testosterone, likely as a precursor for oestradiol synthesis via adipose tissue aromatisation. HI and SHBG showed synergistic inverse associations with total prostate cancer risk with unclear mechanism.

## AUTHOR CONTRIBUTIONS

**Sofia Christakoudi:** Conceptualization (lead); data curation (lead); formal analysis (lead); investigation (equal); methodology (lead); visualization (lead); writing – original draft (lead); writing – review and editing (equal). **Konstantinos K. Tsilidis:** Conceptualization (equal); investigation (equal); methodology (equal); resources (supporting); supervision (equal); visualization (supporting); writing – original draft (supporting); writing – review and editing (equal). **Evangelos Evangelou:** Conceptualization (equal); investigation (equal); methodology (equal); supervision (supporting); visualization (supporting); writing – original draft (supporting); writing – review and editing (equal). **Elio Riboli:** Conceptualization (equal); funding acquisition (lead); investigation (equal); methodology (equal); resources (lead); supervision (lead); writing – original draft (supporting); writing – review and editing (equal).

## FUNDING INFORMATION

This work was supported by the National Institute for Health Research (NIHR) Imperial Biomedical Research Centre (BRC), which provided infrastructure support for the Department of Epidemiology and Biostatistics at Imperial College London (UK). The funder had no role in the design and conduct of the study, the collection, analysis, and interpretation of the data, or the preparation, review, and approval of the manuscript, or in the decision to submit the manuscript for publication.

## CONFLICT OF INTEREST STATEMENT

The authors declare no competing interests.

## ETHICS STATEMENT

This research was conducted according to the principles expressed in the Declaration of Helsinki. The UK Biobank cohort has been approved by the North West Multicenter Research Ethics Committee, UK (Ref: 16/NW/0274). Written informed consent has been obtained from all study participants. The current study was approved by the UK Biobank access management board. Participants who had withdrawn consent by the time of the analysis were excluded from the analysis dataset.

## Supporting information


Appendix S1

## Data Availability

The data supporting the findings of the study are available *to* bona fide researchers upon approval of an application to the UK Biobank (https://www.ukbiobank.ac.uk/enable‐your‐research) and a material transfer agreement.
