# Supplementary material for: Interactions of obesity, body shape, diabetes and sex steroids with respect to prostate cancer risk in the UK Biobank cohort
Source: Cancer Med. 2024 Jan 17;13(3):e6918. doi: 10.1002/cam4.6918 (PMC10905680; doi:10.1002/cam4.6918)
Supplement: Supplementary file 1 — Appendix S1 [file CAM4-13-e6918-s001.pdf]

# Interactions of obesity, body shape, diabetes, and sex steroids with respect to prostate cancer risk in the UK Biobank cohort

Sofia Christakoudi, Konstantinos K. Tsilidis, Evangelos Evangelou, Elio Riboli

Definition of covariates ..... 2

**Supplementary Tables**

Supplementary Table S1 Flow chart of UK Biobank participants in the study ..... 3

Supplementary Table S2 Categories of self-reported medications in men ..... 4

Supplementary Table S3 Rationale for selection of candidate covariates ..... 5

Supplementary Table S4 Characteristics of study participants by BMI and diabetes category ..... 6

Supplementary Table S5 Associations of anthropometric indices and diabetes with prostate cancer risk (sensitivity analyses) ..... 8

**Supplementary Figures**

Supplementary Figure S1 Pairwise associations of candidate covariates with the exposures and with prostate cancer risk ..... 12

Supplementary Figure S2 Associations of total testosterone with prostate cancer risk and interactions of sex steroids and SHBG with ABSI and HI ..... 13

Supplementary Figure S3 Associations of free testosterone and oestradiol with prostate cancer risk (mutual adjustment) ..... 14

**References** ..... 15

**Definition of covariates**

Cited references correspond to the main document.

The following variables were defined as previously described in [8]: age at recruitment (5-year categories for stratification of survival models, continuous for associations of anthropometric indices and diabetes with biomarkers), region of the assessment centre (London, North-West, North-East, Yorkshire and Humber, West Midlands, East Midlands, South-East, South-West, Wales, Scotland), weight change with the year preceding recruitment (weight loss, stable weight, weight gain), smoking status (never smoked, former occasional smoker, former regular smoker, current smoker), alcohol consumption (≤3 times/month, ≤4 times/week, daily), physical activity (less active, moderately active, active), Townsend deprivation index (dataset-specific quintiles with cut-offs: -3.982, -2.861, -1.480, 1.100). The following variables were defined as previously described in [6]: fasting time (0-2 hours, 3-4 hours, ≥5 hours), time of blood collection (morning <12:00, afternoon 12:00 to <16:00, evening ≥16:00 o'clock). Missing values were assigned the median category for the study dataset as follows: weight change (stable weight), smoking status (former occasional smoker), alcohol consumption (≤4 times/week), physical activity (moderately active), Townsend deprivation index (third quintile), fasting time (3-4 hours), time of blood collection (afternoon).

Diabetes status at recruitment was defined similarly to [ref. 17], based on Field [2443-0.0] "*Diabetes diagnosed by doctor*", Question: "*Has a doctor ever told you that you have diabetes?*", Answer: 1 "*Yes*" (for category Yes, n=12,194 in the final dataset), or answer 0 "No" (for category No), considering Answer -1 "*Do not know*" and -3 "*Prefer not to answer*" as missing information. We further re-classified to category Yes participants with self-reported diabetes: codes: 1220 "diabetes", 1222 "type 1 diabetes", or 1223 "type 2 diabetes" in Fields [20002-0.0/33] "*Non-cancer illness code, self-reported*" (further n=92). We additionally re-classified as category Yes participants with self-reported insulin use from Fields [6177-0.0/2] "*Medication for cholesterol, blood pressure or diabetes*", Question "*Do you regularly take any of the following medications? (you can select more than one answer)*", Answer 3 "*Insulin*" (further n=72). We also re-classified as category Yes participants with self-reported antidiabetic drugs from Fields [20003-0/47] "Treatment/medication code" (see list of medication codes in Supplementary Table S2) (further n=28). Last, in addition to [17], we re-classified as category Yes participants with glycated haemoglobin HbA1c≥48 mmol/mol at recruitment (further n=1601). It was not possible to discriminate between type 1 and type 2 diabetes based on the available information. Missing values (n=475) were assigned the median category (No) only for subgroup analyses according to diabetes status and were imputed with multiple imputations for all other analyses.

Family history of cancer was based on three variables, as in [8]: Fields [20107-0.0/9] "Illness of father", Fields [20110-0.0/10] "Illness of mother", and Fields [20111-0.0/11] "Illness of siblings". Category "breast/lung/bowel" was based on Answers: 3 "Lung cancer", 4 "Bowel cancer", 5 "Breast cancer" to any of the three sets of fields, category "prostate" was based on Answer: 13 "Prostate cancer" to any of the three sets of fields, and category No included all remaining participants.

**Supplementary Table S1 Flow chart of UK Biobank participants in the study**

| | Exclusions | Men |
|---|---|---|
| | Total (excluding withdrawals up to the time of analysis): | 229,056 |
| 1. | Ethnic background (restricted to self-reported white) [a] | 13,862 |
| 2. | Anthropometric measurements missing [b] | 1144 |
| 3. | Genetic & self-reported sex mismatch or sex chromosome aneuploidy [a] | 404 |
| 4. | Prevalent cancer at recruitment [a] | 12,270 |
| 5. | Sex steroid treatment [c] | 2874 |
| 6. | Prostate surgery [e] | 2689 |
| | Total excluded (%): | 33,243 (14.5) |
| | Total included (%): | 195,813 (85.5) |

The exclusion criteria were applied sequentially in the displayed order, counting each excluded individual only once.

[a] – for UK Biobank Field names, definition of variables, and definition of prevalent cancer cases see **Supplementary Methods** in [8].

[b] – missing "a body shape index" (ABSI) or hip index (HI). Field names for waist and hip circumferences, weight, and height are listed in **Supplementary Methods** of [8].

[c] – self-reported medication use was based on Fields [20003-0.0…47] "*Treatment/medication code*" (see list of medications in **Supplementary Table S2**).

[e] – self-reported operations were based on Fields [20004-0.0…31] "*Operation code*" (codes correspond to UK Biobank Coding 5) and comprised:

1207 prostate operation
1208 radical prostatectomy
1209 transurethral resection of prostate (turp)

**Supplementary Table S2 Categories of self-reported medications in men**

| Code | Name | Code | Name |
|---|---|---|---|
| **Part A: Antidiabetic drugs used by men (contributed to the definition of diabetes)** | | | |
| 1140857494 | glibornuride | 1140884600 | metformin |
| 1140874646 | glipizide | 1141152590 | glimepiride |
| 1140874652 | minodiab 2.5mg tablet | 1141156984 | amaryl 1mg tablet |
| 1140874674 | tolbutamide | 1141171646 | pioglitazone |
| 1140874686 | glucophage 500mg tablet | 1141171652 | actos 15mg tablet |
| 1140874706 | chlorpropamide | 1141177600 | rosiglitazone |
| 1140874718 | glibenclamide | 1141177606 | avandia 4mg tablet |
| 1140874744 | gliclazide | 1141189090 | rosiglitazone 1mg / metformin 500mg tablet |
| 1140874746 | diamicron 80mg tablet | 1141189094 | avandamet 1mg / 500mg tablet |
| 1140883066 | insulin product | | |
| **Part B: Excluded from the study** | | | |
| **Sex steroids** | | | |
| 1140864196 | climagest 1mg tablet | 1140870232 | megace 40mg tablet |
| 1140864232 | provera 2.5mg tablet | 1140870260 | cyprostat 50mg tablet |
| 1140865136 | yohimbine/pemoline/methyltestosterone | 1140870274 | flutamide |
| 1140866236 | spironolactone | 1140870284 | prostap sr 3.75mg injection (pdr for recon)+diluent+kit |
| 1140866318 | spirolone 25mg tablet | 1140884544 | leuprorelin |
| 1140868406 | conjugated oestrogens | 1140884634 | cyproterone |
| 1140868408 | premarin 625micrograms tablet | 1140884638 | clomiphene |
| 1140868446 | ethinyloestradiol | 1140884726 | nandrolone |
| 1140868456 | oestradiol product | 1140909848 | diethylstilbestrol |
| 1140868460 | progynova 1mg tablet | 1140909920 | gonadotrophin-releasing hormone product |
| 1140868482 | tibolone | 1140909922 | gnrh - gonadotrophin-releasing hormone product |
| 1140868494 | dydrogesterone | 1140910640 | luteine |
| 1140868526 | mesterolone | 1140921100 | triptorelin |
| 1140868532 | testosterone product | 1140922562 | femoston 1/10 tablet |
| 1140868534 | primoteston depot 250mg/1ml oily injection | 1140923738 | femseven 50 patch |
| 1140868536 | restandol 40mg capsule | 1140928222 | andropatch 2.5mg/24hours transdermal patch |
| 1140868538 | sustanon 100 oily injection | 1140928878 | zumenon 1mg tablet |
| 1140868550 | finasteride | 1141151718 | evorel conti patch |
| 1140868588 | progesterone product | 1141157394 | goserelin product |
| 1140868608 | proscar 5mg tablet | 1141165324 | cetrotide 0.25mg injection (pdr for recon)+solvent |
| 1140868628 | humegon 75iu injection (pdr for recon)+solvent | 1141167206 | oestrogel 0.06% gel |
| 1140868968 | danazol | 1141179886 | propecia 1mg tablet |
| 1140868972 | danol 100mg capsule | 1141180766 | novofem tablet |
| 1140869258 | neocon 1/35 tablet | 1141181594 | estriol product |
| 1140869266 | trinovum tablet | 1141181700 | estradiol product |
| 1140869270 | medroxyprogesterone | 1141192000 | dutasteride |
| 1140869352 | norinyl-1 tablet | 1141192004 | avodart 500micrograms capsule |
| 1140870070 | stilboestrol | 1141192344 | cyproterone acetate+ethinylestradiol |
| 1140870164 | tamoxifen | 1141193272 | testogel 50mg gel 5g sachet |
| 1140870182 | emblon 10mg tablet | 1141201718 | nebido 1000mg/4ml solution for injection |
| 1140870194 | goserelin | 1141202030 | estradot 25micrograms patch |
| 1140870196 | zoladex 3.6mg implant | | |

Coding 4 in UK Biobank. The table shows screened medications reported in men. The complete lists of screened medications are the same as in our previous studies [6, 17].

**Supplementary Table S3 Rationale for selection of candidate covariates**

| Factor | Associations with the Exposures | Associations with Prostate Cancer |
|---|---|---|
| Height | To remove residual correlations for allometric anthropometric indices, as discussed in Supplementary Methods of [54]. Sex steroids regulate bone growth in young age [55]. | Highly suggestive evidence for a positive association in an umbrella meta-analysis of prospective observational studies [4]. |
| Weight change | Would reflect different BMI trajectories. | No clear evidence [56]. |
| Smoking | Abdominal obesity in smokers [35, 36]. Higher testosterone in smokers [57]. Smoking facilitates oestrogen deactivation via synthesis of catechol oestrogens [58]. | Lower risk, most pronounced for low-risk prostate cancer from a pooled analysis with 24,731 incident cases [59]. |
| Alcohol | Lower testosterone and SHBG but higher oestradiol for chronic alcohol consumption in men without alcohol use disorder [60]. | Higher risk from a dose-response meta-analysis [61]. |
| Physical activity | Testosterone increases after physical activity [62]. High-intensity physical activity reduces total and visceral fat [63]. | Little evidence [4]. |
| SEP | Low SEP is associated with higher risk of metabolic syndrome [64]. | Higher incidence of low-moderate risk for higher SEP [65]. |
| Fasting time & Time of blood collection | Lower testosterone levels in blood collected in the afternoon [66]. Lower postprandial testosterone levels compared to fasting [67]. | May improve precision of risk estimates, as a way of standardising the conditions of blood collection for biomarker measurements (time of day and fasting time). |

**BMI** – body mass index; **SEP** – socio-economic position.

Pairwise associations of candidate covariates with the exposures and the outcome are shown in **Supplementary Figure S1**.

**Supplementary Table S4 Characteristics of study participants by BMI and diabetes category**

| | **Obese No** BMI<30 kg/m$^2$ | **Obese Yes** BMI≥30 kg/m$^2$ | **Diabetes No** | **Diabetes Yes** |
|---|---|---|---|---|
| Cohort: n (%) | 145,950 (74.5) | 49,863 (25.5) | 181,826 (92.9) | 13,987 (7.1) |
| **Weight change**: n (%) | | | | |
| Weight loss | 20,134 (13.8) | 8294 (16.6) | 24,929 (13.7) | 3499 (25.0) |
| Stable weight | 97,362 (66.7) | 22,919 (46.0) | 113,295 (62.3) | 6986 (49.9) |
| Weight gain | 26,002 (17.8) | 17,729 (35.6) | 40,446 (22.2) | 3285 (23.5) |
| Missing | 2452 (1.7) | 921 (1.8) | 3156 (1.7) | 217 (1.6) |
| **Smoking status**: n (%) | | | | |
| Never smoked | 51,311 (35.2) | 15,816 (31.7) | 63,299 (34.8) | 3828 (27.4) |
| Former occasional | 38,899 (26.7) | 10,547 (21.2) | 46,923 (25.8) | 2523 (18.0) |
| Formal regular | 36,661 (25.1) | 17,655 (35.4) | 48,552 (26.7) | 5764 (41.2) |
| Current smoker | 18,676 (12.8) | 5612 (11.3) | 22,502 (12.4) | 1786 (12.8) |
| Missing | 403 (0.3) | 233 (0.5) | 550 (0.3) | 86 (0.6) |
| **Alcohol**: n (%) | | | | |
| ≤3 times / month | 27,388 (18.8) | 12,385 (24.8) | 35,269 (19.4) | 4504 (32.2) |
| ≤4 times / week | 78,175 (53.6) | 26,448 (53.0) | 98,025 (53.9) | 6598 (47.2) |
| Daily | 40,275 (27.6) | 10,984 (22.0) | 48,393 (26.6) | 2866 (20.5) |
| Missing | 112 (0.1) | 46 (0.1) | 139 (0.1) | 19 (0.1) |
| **Family history**: n (%) | | | | |
| No cancer | 95,275 (65.3) | 32,128 (64.4) | 118,407 (65.1) | 8996 (64.3) |
| Breast, lung, bowel | 39,477 (27.0) | 14,167 (28.4) | 49,569 (27.3) | 4075 (29.1) |
| Prostate | 11,198 (7.7) | 3568 (7.2) | 13,850 (7.6) | 916 (6.5) |
| **Diabetes status**: n (%) | | | | |
| Diabetes No | 139,268 (95.4) | 42,083 (84.4) | 181,351 (99.7) | - |
| Diabetes Yes | 6389 (4.4) | 7598 (15.2) | - | 13,987 (100) |
| Missing | 293 (0.2) | 182 (0.4) | 475 (0.3) | - |
| **Townsend index**: n (%) | | | | |
| Mean (standard deviation) | -1.53 (3.00) | -1.07 (3.17) | -1.47 (3.02) | -0.71 (3.36) |
| Missing: n (%) | 169 (0.1) | 69 (0.1) | 219 (0.1) | 19 (0.1) |
| **Region**: n (%) | | | | |
| London | 17,305 (11.9) | 4956 (9.9) | 20,687 (11.4) | 1574 (11.3) |
| North-West | 23,159 (15.9) | 8705 (17.5) | 29,389 (16.2) | 2475 (17.7) |
| North-East | 17,253 (11.8) | 6597 (13.2) | 22,170 (12.2) | 1680 (12.0) |
| Yorkshire Humber | 22,254 (15.2) | 7655 (15.4) | 27,782 (15.3) | 2127 (15.2) |
| West Midlands | 13,228 (9.1) | 4971 (10.0) | 16,777 (9.2) | 1422 (10.2) |
| East Midlands | 10,104 (6.9) | 3401 (6.8) | 12,508 (6.9) | 997 (7.1) |
| South-East | 13,256 (9.1) | 3558 (7.1) | 15,824 (8.7) | 990 (7.1) |
| South-West | 13,023 (8.9) | 3878 (7.8) | 15,841 (8.7) | 1060 (7.6) |
| Wales | 5,844 (4.0) | 2521 (5.1) | 7686 (4.2) | 679 (4.9) |
| Scotland | 10,524 (7.2) | 3621 (7.3) | 13,162 (7.2) | 983 (7.0) |
| **Time of blood collection**: n (%) | | | | |
| <12:00 | 39,128 (26.8) | 12,905 (25.9) | 48,561 (26.7) | 3472 (24.8) |
| 12:00 to <16:00 | 55,710 (38.2) | 19,227 (38.6) | 69,003 (38.0) | 5934 (42.4) |
| ≥16:00 | 50,413 (34.5) | 17,355 (34.8) | 63,284 (34.8) | 4484 (32.1) |
| Missing | 699 (0.5) | 376 (0.8) | 978 (0.5) | 97 (0.7) |
| **Biomarker availability**: n (%) | | | | |
| E2 | 128,312 (87.9) | 43,546 (87.3) | 159,556 (87.8) | 12,302 (88.0) |
| T & fT & SHBG | 125,288 (85.8) | 42,803 (85.8) | 156,141 (85.9) | 11,950 (85.4) |
| T & fT & SHBG & E2 | 116,765 (80.0) | 39,860 (79.9) | 145,476 (80.0) | 11,149 (79.7) |
| HbA1c | 137,131 (94.0) | 46,664 (93.6) | 170,574 (93.8) | 13,221 (94.5) |

| | Obese No BMI<30 kg/m$^2$ | Obese Yes BMI≥30 kg/m$^2$ | Diabetes No | Diabetes Yes |
|---|---|---|---|---|
| **Cross-classification cohorts**: n (%) | | | | |
| BMI<30 kg/m$^2$: Low | | | 139,561 (76.8) | 6389 (45.7) |
| BMI≥30 kg/m$^2$: High | | | 42,265 (23.2) | 7598 (54.3) |
| ABSI<79.8: Low | 75,308 (51.6) | 22,597 (45.3) | 93,042 (51.2) | 4863 (34.8) |
| ABSI≥79.8: High | 70,642 (48.4) | 27,266 (54.7) | 88,784 (48.8) | 9124 (65.2) |
| HI <49.1: Low | 72,653 (49.8) | 25,252 (50.6) | 90,324 (49.7) | 7581 (54.2) |
| HI ≥49.1: High | 73,297 (50.2) | 24,611 (49.4) | 91,502 (50.3) | 6406 (45.8) |
| TT <11.6 nmol/L: Low | 55,416 (44.2) | 28,628 (66.9) | 75,804 (48.5) | 8240 (69.0) |
| TT ≥11.6 nmol/L: High | 69,872 (55.8) | 14,175 (33.1) | 80,337 (51.5) | 3710 (31.0) |
| FT <243 pmol/L: Low | 57,980 (46.3) | 26,065 (60.9) | 76,023 (48.7) | 8022 (67.1) |
| FT ≥243 pmol/L: High | 67,308 (53.7) | 16,738 (39.1) | 80,118 (51.3) | 3928 (32.9) |
| SHBG <37.1 nmol/L: Low | 56,262 (44.9) | 27,763 (64.9) | 76,693 (49.1) | 7332 (61.4) |
| SHBG ≥37.1 nmol/L: High | 69,026 (55.1) | 15,040 (35.1) | 79,448 (50.9) | 4618 (38.6) |
| Oestradiol <175 pmol/L: Low | 117,424 (91.5) | 38,996 (89.6) | 145,314 (91.1) | 11,106 (90.3) |
| Oestradiol ≥175 pmol/L: High | 10,888 (8.5) | 4550 (10.4) | 14,242 (8.9) | 1196 (9.7) |
| **BMI in cross-classification groups** (kg/m$^2$): median (2.5$^{th}$ – 97.5$^{th}$ centile) | | | | |
| BMI<30 kg/m$^2$: Low | | | 26.1 (20.6-29.7) | 27.3 (21.6-29.9) |
| BMI≥30 kg/m$^2$: High | | | 32.2 (30.1-41.7) | 33.8 (30.2-46.1) |
| ABSI<79.8: Low | 26.1 (20.7-29.7) | 32.2 (30.1-42.1) | 27.0 (21.0-36.4) | 30.2 (22.4-43.6) |
| ABSI≥79.8: High | 26.2 (20.7-29.8) | 32.5 (30.1-43.0) | 27.3 (21.0-37.4) | 30.7 (22.9-44.0) |
| HI <49.1: Low | 26.4 (21.1-29.8) | 32.1 (30.1-40.1) | 27.3 (21.4-35.9) | 30.2 (23.0-40.5) |
| HI ≥49.1: High | 25.9 (20.4-29.7) | 32.7 (30.1-44.6) | 26.9 (20.7-37.9) | 31.1 (22.4-46.2) |
| TT <11.6 nmol/L: Low | 26.7 (21.4-29.8) | 32.6 (30.1-43.6) | 28.1 (21.8-38.4) | 31.2 (23.6-45.2) |
| TT ≥11.6 nmol/L: High | 25.7 (20.3-29.7) | 31.9 (30.1-40.1) | 26.3 (20.5-34.7) | 29.0 (21.6-40.3) |
| FT <243 pmol/L: Low | 26.4 (20.6-29.8) | 32.6 (30.1-43.8) | 27.6 (21.1-38.2) | 31.0 (23.0-45.1) |
| FT ≥243 pmol/L: High | 26.0 (20.7-29.7) | 32.0 (30.1-40.4) | 26.7 (20.9-35.3) | 29.6 (22.3-41.2) |
| SHBG <37.1 nmol/L: Low | 26.8 (22.0-29.8) | 32.5 (30.1-43.0) | 28.1 (22.3-37.9) | 31.3 (24.0-45.0) |
| SHBG ≥37.1 nmol/L: High | 25.6 (20.1-29.6) | 32.1 (30.1-41.9) | 26.2 (20.3-35.4) | 29.3 (21.7-42.2) |
| Oestradiol <175 pmol/L: Low | 26.2 (20.7-29.7) | 32.3 (30.1-42.2) | 27.1 (21.0-36.6) | 30.4 (22.8-43.5) |
| Oestradiol ≥175 pmol/L: High | 26.2 (20.7-29.8) | 32.9 (30.1-45.5) | 27.4 (21.0-39.3) | 31.2 (22.7-45.9) |
| **ABSI in cross-classification groups**: median (2.5$^{th}$ – 97.5$^{th}$ centile) | | | | |
| ABSI <79.8: Low | 77.0 (70.3-79.6) | 77.3 (71.0-79.7) | 77.0 (70.4-79.6) | 77.6 (71.5-79.7) |
| ABSI ≥79.8: High | 82.4 (79.9-89.1) | 82.6 (79.9-88.9) | 82.4 (79.9-88.9) | 83.1 (79.9-89.9) |
| **HI in cross-classification groups**: median (2.5$^{th}$ – 97.5$^{th}$ centile) | | | | |
| HI <49.1: Low | 48.1 (45.1-49.1) | 48.0 (44.9-49.1) | 48.1 (45.1-49.1) | 47.9 (44.6-49.1) |
| HI ≥49.1: High | 50.1 (49.2-52.6) | 50.3 (49.2-54.7) | 50.1 (49.2-53.0) | 50.4 (49.2-55.4) |
| **Biomarker levels in cross-classification groups**: median (2.5$^{th}$ – 97.5$^{th}$ centile) | | | | |
| TT <11.6 nmol/L: Low | 9.7 (5.7-11.5) | 9.0 (4.8-11.5) | 9.5 (5.4-11.5) | 8.7 (4.4-11.5) |
| TT ≥11.6 nmol/L: High | 14.3 (11.8-22.0) | 13.6 (11.7-20.3) | 14.2 (11.7-21.7) | 13.7 (11.7-21.5) |
| FT <243 pmol/L: Low | 207 (126-241) | 199 (115-240) | 206 (124-241) | 194 (109-240) |
| FT ≥243 pmol/L: High | 287 (245-421) | 281 (244-409) | 286 (245-419) | 277 (244-395) |
| SHBG <37.1 nmol/L: Low | 28.8 (14.2-36.7) | 26.4 (11.9-36.4) | 28.3 (13.5-36.6) | 26.1 (10.9-36.5) |
| SHBG ≥37.1 nmol/L: High | 48.9 (37.6-89.1) | 45.7 (37.4-81.7) | 48.3 (37.5-87.8) | 47.5 (37.4-98.0) |

**n (%)** – number of participants (percentage from total overall for cohort or total per column otherwise); **ABSI** – a body shape index; **BMI** – body mass index; **FT** – free testosterone; **HI** – hip index; **SHBG** – sex hormone binding globulin; **TT** – total testosterone. Participant characteristics were compared between obesity and diabetes status groups with t-test for Townsend deprivation index and $\chi^2$-test for categorical variables. All differences were significant at p<0.0001, except biomarker availability.

## Supplementary Table S5 Associations of anthropometric indices and diabetes with prostate cancer risk (sensitivity analyses)

| **Groups of men** | | **Overall** | | | **Obese** | | | **Diabetes Yes** | |
|---|---|---|---|---|---|---|---|---|---|
| number all (number ≥2 years follow-up) | | n=195,813 (191, 882) | | | n=49,863 (48,815) | | | n=13,987 (13,530) | |
| **Model adjustments** | cases | HR (95% CI) | p-value | cases | HR (95% CI) | p-value | cases | HR (95% CI) | p-value |
| **BMI** (per one SD) [#] | | | | | [#] | | | | |
| Minimally adjusted [a] | 9417 | 0.931 (0.911 to 0.951) | <0.0001 | 2100 | 0.858 (0.804 to 0.915) | <0.0001 | | | |
| + Covariates [b] | 9417 | 0.946 (0.925 to 0.968) | <0.0001 | 2100 | 0.877 (0.822 to 0.937) | <0.0001 | | | |
| adjustment difference (covariates) | | 0.015 | | | **0.020** | | | | |
| + Covariates + Diabetes [c] | 9417 | 0.959 (0.937 to 0.982) | 0.0005 | 2100 | 0.905 (0.847 to 0.967) | 0.003 | 592 | 0.832 (0.768 to 0.902) | <0.0001 |
| adjustment difference (covariates + diabetes) | | **0.029** | | | **0.047** | | | | |
| ≥2 years follow-up (Covariates+Diabetes) [d] | 8179 | 0.959 (0.935 to 0.983) | 0.0009 | 1810 | 0.899 (0.838 to 0.966) | 0.003 | 511 | 0.837 (0.768 to 0.913) | <0.0001 |
| lag difference | | -0.001 | | | -0.006 | | | 0.005 | |
| **ABSI** (per one SD) | | | | | | | | | |
| Minimally adjusted [a] | 9417 | 1.003 (0.982 to 1.025) | 0.770 | 2100 | 1.070 (1.020 to 1.121) | 0.005 | | | |
| + Covariates [b] | 9417 | 1.007 (0.985 to 1.030) | 0.520 | 2100 | 1.069 (1.018 to 1.122) | 0.007 | | | |
| adjustment difference (covariates) | | 0.004 | | | -0.001 | | | | |
| + Covariates + Diabetes [c] | 9417 | 1.012 (0.990 to 1.034) | 0.303 | 2100 | 1.081 (1.030 to 1.135) | 0.002 | 592 | 1.114 (1.021 to 1.216) | 0.015 |
| adjustment difference (covariates + diabetes) | | 0.008 | | | 0.012 | | | | |
| ≥2 years follow-up (Covariates+Diabetes) [d] | 8179 | 1.009 (0.986 to 1.034) | 0.445 | 1810 | 1.073 (1.018 to 1.131) | 0.009 | 511 | 1.105 (1.006 to 1.214) | 0.038 |
| lag difference | | -0.002 | | | -0.009 | | | -0.009 | |
| **HI** (per one SD) | | | | | | | | | |
| Minimally adjusted [a] | 9417 | 0.982 (0.962 to 1.003) | 0.090 | 2100 | 1.002 (0.963 to 1.042) | 0.927 | | | |
| + Covariates [b] | 9417 | 0.969 (0.949 to 0.990) | 0.004 | 2100 | 0.994 (0.955 to 1.034) | 0.767 | | | |
| adjustment difference (covariates) | | -0.013 | | | -0.008 | | | | |
| + Covariates + Diabetes [c] | 9417 | 0.967 (0.947 to 0.988) | 0.002 | 2100 | 0.988 (0.949 to 1.028) | 0.541 | 592 | 0.950 (0.882 to 1.024) | 0.180 |
| adjustment difference (covariates + diabetes) | | -0.015 | | | -0.014 | | | | |
| ≥2 years follow-up (Covariates+Diabetes) [d] | 8179 | 0.968 (0.947 to 0.991) | 0.006 | 1810 | 0.991 (0.949 to 1.034) | 0.668 | 511 | 0.954 (0.880 to 1.034) | 0.250 |
| lag difference | | 0.002 | | | 0.003 | | | 0.004 | |
| **Diabetes** (Yes vs No) | | | | | | | | | |
| Covariates + Diabetes [c] | 9417 | 0.772 (0.709 to 0.842) | <0.0001 | 2100 | 0.691 (0.604 to 0.790) | <0.0001 | | | |
| ≥2 years follow-up (Covariates+Diabetes) [d] | 8179 | 0.783 (0.714 to 0.859) | <0.0001 | 1810 | 0.706 (0.611 to 0.816) | <0.0001 | | | |
| lag difference | | 0.010 | | | 0.015 | | | | |

| Groups of men | Overall | | | Obese | | | Diabetes Yes | | |
|---|---|---|---|---|---|---|---|---|---|
| **Model adjustments** | cases | HR (95% CI) | p-value | cases | HR (95% CI) | p-value | cases | HR (95% CI) | p-value |
| **+ TT or + FT + SHBG** | | n=168,091 | | | n=42,803 | | | n=11,950 | |
| **BMI** (Covariates+Diabetes) [e] | 8106 | 0.966 (0.942 to 0.990) | 0.006 | 1816 | 0.920 (0.857 to 0.987) | 0.021 | 507 | 0.837 (0.768 to 0.913) | <0.0001 |
| **BMI** (Covariates+Diabetes +TT) [f] | 8106 | 0.968 (0.943 to 0.993) | 0.013 | 1816 | 0.918 (0.855 to 0.986) | 0.019 | 507 | 0.862 (0.789 to 0.942) | 0.001 |
| adjustment difference (TT) | | 0.002 | | | -0.002 | | | **0.024** | |
| **BMI** (Covariates+Diabetes +FT+SHBG) [f] | 8106 | 0.951 (0.927 to 0.977) | 0.0002 | 1816 | 0.921 (0.857 to 0.989) | 0.024 | 507 | 0.847 (0.774 to 0.927) | 0.0003 |
| adjustment difference (FT+SHBG) | | -0.014 | | | 0.001 | | | 0.010 | |
| **ABSI** (Covariates+Diabetes) [e] | 8106 | 1.012 (0.989 to 1.037) | 0.312 | 1816 | 1.086 (1.031 to 1.144) | 0.002 | 507 | 1.142 (1.039 to 1.254) | 0.006 |
| **ABSI** (Covariates+Diabetes +TT) [f] | 8106 | 1.013 (0.989 to 1.037) | 0.288 | 1816 | 1.085 (1.030 to 1.143) | 0.002 | 507 | 1.155 (1.051 to 1.269) | 0.003 |
| adjustment difference (TT) | | 0.001 | | | -0.001 | | | 0.013 | |
| **ABSI** (Covariates+Diabetes +FT+SHBG) [f] | 8106 | 1.010 (0.986 to 1.035) | 0.403 | 1816 | 1.088 (1.033 to 1.146) | 0.002 | 507 | 1.148 (1.044 to 1.262) | 0.004 |
| adjustment difference (fT+SHBG) | | -0.002 | | | 0.002 | | | 0.006 | |
| **HI** (Covariates+Diabetes) [e] | 8106 | 0.968 (0.946 to 0.990) | 0.005 | 1816 | 0.987 (0.945 to 1.030) | 0.545 | 507 | 0.952 (0.878 to 1.032) | 0.235 |
| **HI** (Covariates+Diabetes +TT) [f] | 8106 | 0.967 (0.946 to 0.990) | 0.005 | 1816 | 0.987 (0.945 to 1.030) | 0.543 | 507 | 0.949 (0.875 to 1.029) | 0.206 |
| adjustment difference (TT) | | 0 | | | 0 | | | -0.003 | |
| **HI** (Covariates+Diabetes +FT+SHBG) [f] | 8106 | 0.976 (0.954 to 0.999) | 0.043 | 1816 | 0.992 (0.950 to 1.036) | 0.713 | 507 | 0.964 (0.888 to 1.046) | 0.377 |
| adjustment difference (FT+SHBG) | | 0.009 | | | 0.005 | | | 0.012 | |
| **Diabetes** (Covariates+Diabetes) [e] | 8106 | 0.769 (0.701 to 0.843) | <0.0001 | 1816 | 0.693 (0.600 to 0.801) | <0.0001 | 507 | - | |
| **Diabetes** (Covariates+Diabetes +TT) [f] | 8106 | 0.770 (0.702 to 0.845) | <0.0001 | 1816 | 0.692 (0.598 to 0.800) | <0.0001 | 507 | - | |
| adjustment difference (TT) | | 0.002 | | | -0.002 | | | | |
| **Diabetes** (Covariates+Diabetes+FT+SHBG) [f] | 8106 | 0.766 (0.697 to 0.840) | <0.0001 | 1816 | 0.683 (0.591 to 0.790) | <0.0001 | 507 | - | |
| adjustment difference (FT+SHBG) | | -0.003 | | | -0.010 | | | | |
| **+ Oestradiol** | | n=171,858 | | | n=43,546 | | | n=12,302 | |
| **BMI** (Covariates+Diabetes) [g] | 8282 | 0.967 (0.943 to 0.991) | 0.008 | 1853 | 0.917 (0.855 to 0.984) | 0.016 | 512 | 0.851 (0.781 to 0.926) | 0.0002 |
| **BMI** (Covariates+Diabetes +E2) [h] | 8282 | 0.967 (0.944 to 0.992) | 0.009 | 1853 | 0.922 (0.860 to 0.989) | 0.024 | 512 | 0.851 (0.782 to 0.927) | 0.0002 |
| adjustment difference (E2) | | 0 | | | 0.005 | | | 0.001 | |
| **ABSI** (Covariates+Diabetes) [g] | 8282 | 1.006 (0.983 to 1.030) | 0.594 | 1853 | 1.080 (1.026 to 1.137) | 0.003 | 512 | 1.150 (1.047 to 1.262) | 0.003 |
| **ABSI** (Covariates+Diabetes +E2) [h] | 8282 | 1.006 (0.983 to 1.030) | 0.600 | 1853 | 1.079 (1.025 to 1.136) | 0.004 | 512 | 1.149 (1.047 to 1.262) | 0.003 |
| adjustment difference (E2) | | 0 | | | 0 | | | 0 | |

| Groups of men | Overall | | | Obese | | | Diabetes Yes | | |
|---|---|---|---|---|---|---|---|---|---|
| **Model adjustments** | cases | HR (95% CI) | p-value | cases | HR (95% CI) | p-value | cases | HR (95% CI) | p-value |
| **+ Oestradiol** *(continued)* | | n=171,858 | | | n=43,546 | | | n=12,302 | |
| **HI** (Covariates+Diabetes) [g] | 8282 | 0.969 (0.947 to 0.991) | 0.006 | 1853 | 0.984 (0.943 to 1.027) | 0.460 | 512 | 0.955 (0.882 to 1.035) | 0.261 |
| **HI** (Covariates+Diabetes +E2) [h] | 8282 | 0.969 (0.947 to 0.991) | 0.006 | 1853 | 0.985 (0.944 to 1.028) | 0.481 | 512 | 0.956 (0.883 to 1.035) | 0.266 |
| adjustment difference (E2) | | 0 | | | 0.001 | | | 0 | |
| **Diabetes** (Covariates+Diabetes) [g] | 8282 | 0.751 (0.685 to 0.824) | <0.0001 | 1853 | 0.682 (0.592 to 0.787) | <0.0001 | 512 | - | |
| **Diabetes** (Covariates+Diabetes +E2) [h] | 8282 | 0.751 (0.685 to 0.823) | <0.0001 | 1853 | 0.680 (0.590 to 0.785) | <0.0001 | 512 | - | |
| adjustment difference (E2) | | 0 | | | -0.002 | | | | |
| **+ HbA1c (men without diabetes)** | | n=170,135 | | | n=39,314 | | | | |
| **BMI** (Covariates) [i] | 8269 | 0.972 (0.947 to 0.997) | 0.026 | 1720 | 0.924 (0.857 to 0.997) | 0.040 | | - | |
| **BMI** (Covariates +HbA1c) [j] | 8269 | 0.974 (0.950 to 1.000) | 0.046 | 1720 | 0.929 (0.861 to 1.002) | 0.055 | | - | |
| adjustment difference (HbA1c) | | 0.003 | | | 0.004 | | | | |
| **ABSI** (Covariates) [i] | 8269 | 1.007 (0.983 to 1.031) | 0.585 | 1720 | 1.069 (1.012 to 1.128) | 0.016 | | - | |
| **ABSI** (Covariates +HbA1c) [j] | 8269 | 1.007 (0.984 to 1.031) | 0.547 | 1720 | 1.070 (1.014 to 1.130) | 0.014 | | - | |
| adjustment difference (HbA1c) | | 0.001 | | | 0.002 | | | | |
| **HI** (Covariates) [i] | 8269 | 0.972 (0.950 to 0.994) | 0.015 | 1720 | 1.001 (0.956 to 1.047) | 0.971 | | - | |
| **HI** (Covariates +HbA1c) [j] | 8269 | 0.971 (0.949 to 0.994) | 0.013 | 1720 | 1.000 (0.955 to 1.046) | 0.990 | | - | |
| adjustment difference (HbA1c) | | -0.001 | | | -0.001 | | | | |
| **HbA1c** (Covariates) per one SD [j £] | 8269 | 0.983 (0.961 to 1.006) | 0.143 | 1720 | 0.974 (0.928 to 1.022) | 0.280 | | £ | |

**ABSI** – a body shape index; **BMI** – body mass index; **cases** – number of prostate cancer cases per group; **CI** – confidence interval; **E2** – oestradiol; **FT** – free testosterone; **HbA1c** – glycated haemoglobin; **HI** – hip index; **HR** – hazard ratio; **Obese** – BMI≥30 kg/m$^2$; **p-value** – Wald test for the individual term; **SD** – standard deviation; **SHBG** – sex hormone binding globulin; **TT** – total testosterone.

[a] – Cox proportional hazards models, including BMI, ABSI, HI and height (z-scores, value minus mean divided by SD), stratified by age at recruitment;

[b] – like [a], additionally stratified by region of the assessment centre and family history of prostate cancer, and additionally adjusted for recent weight change, smoking status, alcohol consumption, Townsend deprivation index, and time of blood collection;

[c] – like [b], additionally including diabetes status (main models);

[d] – like [c] but in men with ≥2 years of follow-up;

[e] – like [c] but in men with available TT, FT, and SHBG;

[f] – like [e] but additionally including either TT individually or jointly FT and SHBG (z-scores);

[g] – like [c] but in men with available oestradiol measurement;

[h] – like [g] but additionally including oestradiol (detected no/yes);

[i] – like [c] but in men with available HbA1c and without known diabetes;

[j] – like [i] but additionally including HbA1c (z-scores).

**adjustment difference** – differences in HR estimates between the model adjusted only for covariates or the fully adjusted model (covariates + diabetes) and the minimally adjusted model, or between the models with and without biomarkers.

**lag difference** – differences in HR estimates between the model with ≥2 years of follow-up and the fully adjusted model (covariates + diabetes).

[#] associations with BMI (per one SD) in men with BMI<25 kg/$m^2$ (n=42,011):

$HR_{per\_SD}$=1.217 (1.076 to 1.376); p=0.002 for the minimally adjusted model;

$HR_{per\_SD}$=1.185 (1.046 to 1.343); p=0.008 for the model adjusted only for covariates (adjustment difference **-0.032**);

$HR_{per\_SD}$=1.187 (1.047 to 1.345); p=0.007 for the fully adjusted model (adjustment difference **-0.030**);

$HR_{per\_SD}$=1.182 (1.034 to 1.352); p=0.015 for ≥2 years follow-up (lag difference -0.005).

[£] association with HbA1c in men with available HbA1c<48 and without known diabetes (n=170,135):

$HR_{pre\text{-}diabetes}$=0.968 (0.864 to 1.085); p=0.578 for HbA1c≥42 to <48 mmol/mol (pre-diabetes, 318 cases) vs HbA1c<42 mmol/mol (7951 cases).

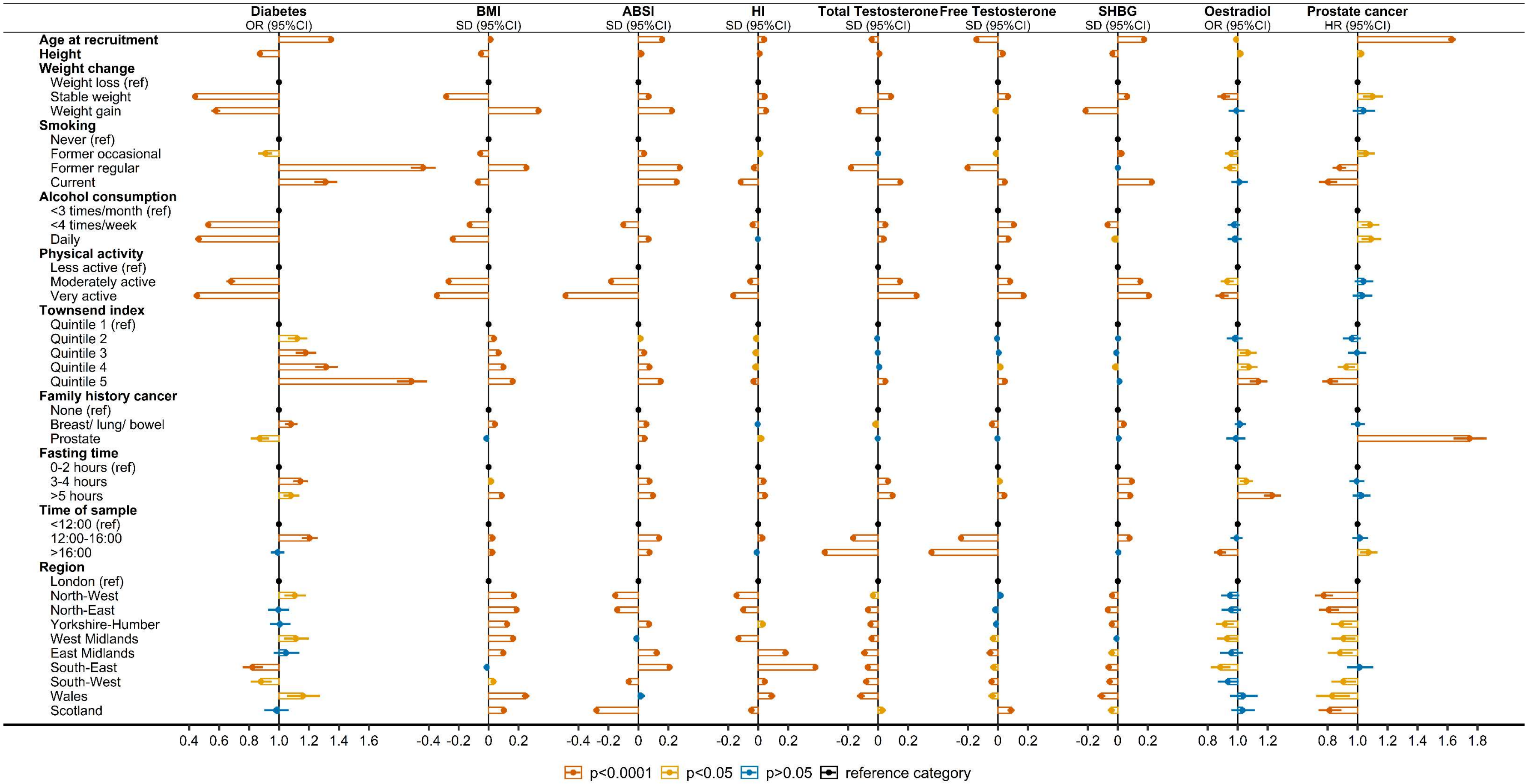


**Supplementary Figure S1 Pairwise associations of candidate covariates with the exposures and with prostate cancer risk**

**ABSI** – a body shape index; **BMI** – body mass index; **CI** – confidence interval; **HR** – hazard ratio; **OR** – odds ratio; **SD** – standard deviation difference compared to the reference category, or standard deviation change per 5 years increase in age at recruitment or per 5 cm increase in height; **SHBG** – sex hormone binding globulin. Estimates from liner regression models (SD), or logistic regression models (OR), or Cox proportional hazards models (HR, timescale age for all covariates, except person years when examining age at recruitment as a covariate), including each exposure specified in the header or prostate cancer as the dependent variable and each potential candidate covariate individually as the independent variable.

| Category | cases | rate | HR (95% CI) | p-value |
|---|---|---|---|---|
| **Total Testosterone** (per one SD) | | | | |
| Overall | 8104 | | 1.008 (0.984 to 1.032) | 0.525 |
| **Obese** | | | | |
| No (NW) | 2068 | | 1.046 (0.996 to 1.098) | 0.072 |
| No (OW) | 4220 | | 1.000 (0.967 to 1.033) | 0.984 |
| Yes | 1816 | | 0.991 (0.946 to 1.038) | 0.694 |
| MI | | | 1.004 (0.983 to 1.025) | 0.712 |
| Low-No | 2867 | 5025 | 1.159 (1.057 to 1.271) | 0.002 |
| High-No | 3421 | 4742 | 1.177 (1.075 to 1.290) | 0.0005 |
| Low-Yes | 1267 | 4345 | 1.095 (0.990 to 1.210) | 0.078 |
| High-Yes | 549 | 3788 | reference | |
| RERI | | | -0.113 (-0.238 to 0.013) | 0.078 |
| AR | | | -0.097 (-0.201 to 0.006) | 0.066 |
| SI | | | 0.585 (0.396 to 0.865) | 0.007 |
| **Diabetes** | | | | |
| No | 7598 | | 1.000 (0.976 to 1.025) | 0.981 |
| Yes | 506 | | 1.116 (1.022 to 1.218) | 0.014 |
| MI | | | 1.130 (1.037 to 1.231) | 0.005 |
| Low-No | 3794 | 4858 | 1.352 (1.207 to 1.513) | <0.0001 |
| High-No | 3804 | 4583 | 1.333 (1.188 to 1.496) | <0.0001 |
| Low-Yes | 340 | 4194 | reference | |
| High-Yes | 166 | 4579 | 1.108 (0.920 to 1.335) | 0.280 |
| RERI | | | -0.126 (-0.342 to 0.089) | 0.251 |
| AR | | | -0.095 (-0.252 to 0.062) | 0.237 |
| SI | | | 0.725 (0.473 to 1.111) | 0.140 |
| **MI with ABSI** | | | | |
| Total Testosterone | | | 0.992 (0.971 to 1.014) | 0.480 |
| Free Testosterone | | | 0.996 (0.973 to 1.019) | 0.709 |
| SHBG | | | 0.982 (0.959 to 1.004) | 0.114 |
| Oestradiol | | | 1.026 (0.947 to 1.110) | 0.532 |
| **MI with HI** | | | | |
| Total Testosterone | | | 0.981 (0.961 to 1.001) | 0.060 |
| Free Testosterone | | | 0.984 (0.964 to 1.005) | 0.138 |
| SHBG | | | 0.974 (0.953 to 0.997) | 0.024 |
| Oestradiol | | | 0.968 (0.897 to 1.045) | 0.405 |

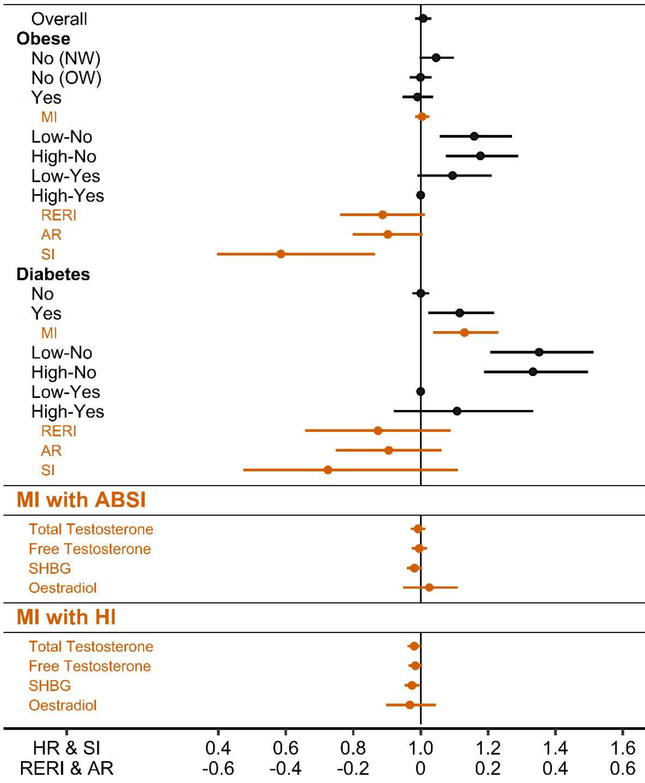


**Supplementary Figure S2 Associations of total testosterone with prostate cancer risk and interactions of sex steroids and SHBG with ABSI and HI**

**ABSI** – a body shape index; **AP** – attributable proportion due to interaction; **cases** – number of incident prostate cancer cases per group; **CI** – confidence interval; **HI** – hip index; **HR** – hazard ratio; **MI** – multiplicative interaction term of each biomarker individually (z-scores or no/yes for oestradiol≥175 pmol/L) with BMI, ABSI, or HI on a continuous scale (z-scores) or with diabetes (no/yes); **NW** – BMI<25 kg/m$^2$; **OW** – BMI≥25 to <30 kg/m$^2$; **Obese** – BMI≥30 kg/m$^2$; **rate** – numer of incident prostate cancer cases per 1,000,000 person years of follow-up per group; **RERI** – relative excess risk from interaction; **SD** – standard deviation; **SHBG** – sex hormone binding globulin; **SI** – synergy index.

Estimates from Cox proportional hazards models in men overall with available total and free testosterone and SHBG measurements (n=168,091), including total testosterone (z-scores, value minus mean divided by SD, following log-transformation) as exposure, stratified by age at recruitment, region of the assessment centre, and family history of prostate cancer, and adjusted for diabetes status, BMI, ABSI, HI, height (z-scores), recent weight change, smoking status, alcohol consumption, Townsend deprivation index, and time of blood collection.

**Low/High–No/Yes** – groups of men according to a cross-classification of total testosterone (low/high), dichotomised at the median (≥11.6 nmol/L), with either obese or diabetes (no/yes).

Associations and interactions with free testosterone, oestradiol, and SHBG are shown in **Figure 2** in the main document.

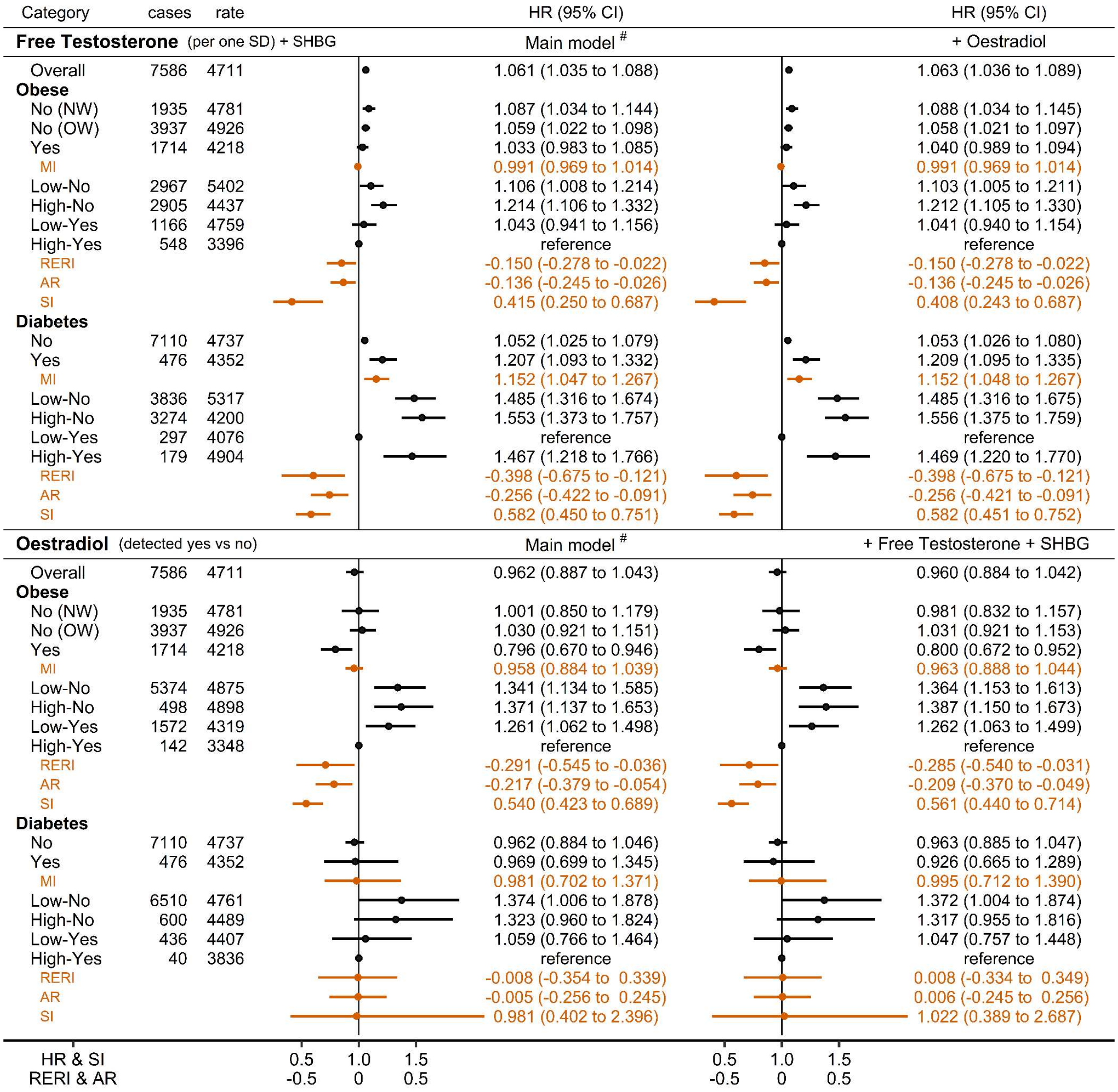


| Category | cases | rate | HR (95% CI) | HR (95% CI) |
|---|---|---|---|---|
| **Free Testosterone** (per one SD) + SHBG | | | Main model # | + Oestradiol |
| Overall | 7586 | 4711 | 1.061 (1.035 to 1.088) | 1.063 (1.036 to 1.089) |
| **Obese** | | | | |
| No (NW) | 1935 | 4781 | 1.087 (1.034 to 1.144) | 1.088 (1.034 to 1.145) |
| No (OW) | 3937 | 4926 | 1.059 (1.022 to 1.098) | 1.058 (1.021 to 1.097) |
| Yes | 1714 | 4218 | 1.033 (0.983 to 1.085) | 1.040 (0.989 to 1.094) |
| MI | | | 0.991 (0.969 to 1.014) | 0.991 (0.969 to 1.014) |
| Low-No | 2967 | 5402 | 1.106 (1.008 to 1.214) | 1.103 (1.005 to 1.211) |
| High-No | 2905 | 4437 | 1.214 (1.106 to 1.332) | 1.212 (1.105 to 1.330) |
| Low-Yes | 1166 | 4759 | 1.043 (0.941 to 1.156) | 1.041 (0.940 to 1.154) |
| High-Yes | 548 | 3396 | reference | reference |
| RERI | | | -0.150 (-0.278 to -0.022) | -0.150 (-0.278 to -0.022) |
| AR | | | -0.136 (-0.245 to -0.026) | -0.136 (-0.245 to -0.026) |
| SI | | | 0.415 (0.250 to 0.687) | 0.408 (0.243 to 0.687) |
| **Diabetes** | | | | |
| No | 7110 | 4737 | 1.052 (1.025 to 1.079) | 1.053 (1.026 to 1.080) |
| Yes | 476 | 4352 | 1.207 (1.093 to 1.332) | 1.209 (1.095 to 1.335) |
| MI | | | 1.152 (1.047 to 1.267) | 1.152 (1.048 to 1.267) |
| Low-No | 3836 | 5317 | 1.485 (1.316 to 1.674) | 1.485 (1.316 to 1.675) |
| High-No | 3274 | 4200 | 1.553 (1.373 to 1.757) | 1.556 (1.375 to 1.759) |
| Low-Yes | 297 | 4076 | reference | reference |
| High-Yes | 179 | 4904 | 1.467 (1.218 to 1.766) | 1.469 (1.220 to 1.770) |
| RERI | | | -0.398 (-0.675 to -0.121) | -0.398 (-0.675 to -0.121) |
| AR | | | -0.256 (-0.422 to -0.091) | -0.256 (-0.421 to -0.091) |
| SI | | | 0.582 (0.450 to 0.751) | 0.582 (0.451 to 0.752) |
| **Oestradiol** (detected yes vs no) | | | Main model # | + Free Testosterone + SHBG |
| Overall | 7586 | 4711 | 0.962 (0.887 to 1.043) | 0.960 (0.884 to 1.042) |
| **Obese** | | | | |
| No (NW) | 1935 | 4781 | 1.001 (0.850 to 1.179) | 0.981 (0.832 to 1.157) |
| No (OW) | 3937 | 4926 | 1.030 (0.921 to 1.151) | 1.031 (0.921 to 1.153) |
| Yes | 1714 | 4218 | 0.796 (0.670 to 0.946) | 0.800 (0.672 to 0.952) |
| MI | | | 0.958 (0.884 to 1.039) | 0.963 (0.888 to 1.044) |
| Low-No | 5374 | 4875 | 1.341 (1.134 to 1.585) | 1.364 (1.153 to 1.613) |
| High-No | 498 | 4898 | 1.371 (1.137 to 1.653) | 1.387 (1.150 to 1.673) |
| Low-Yes | 1572 | 4319 | 1.261 (1.062 to 1.498) | 1.262 (1.063 to 1.499) |
| High-Yes | 142 | 3348 | reference | reference |
| RERI | | | -0.291 (-0.545 to -0.036) | -0.285 (-0.540 to -0.031) |
| AR | | | -0.217 (-0.379 to -0.054) | -0.209 (-0.370 to -0.049) |
| SI | | | 0.540 (0.423 to 0.689) | 0.561 (0.440 to 0.714) |
| **Diabetes** | | | | |
| No | 7110 | 4737 | 0.962 (0.884 to 1.046) | 0.963 (0.885 to 1.047) |
| Yes | 476 | 4352 | 0.969 (0.699 to 1.345) | 0.926 (0.665 to 1.289) |
| MI | | | 0.981 (0.702 to 1.371) | 0.995 (0.712 to 1.390) |
| Low-No | 6510 | 4761 | 1.374 (1.006 to 1.878) | 1.372 (1.004 to 1.874) |
| High-No | 600 | 4489 | 1.323 (0.960 to 1.824) | 1.317 (0.955 to 1.816) |
| Low-Yes | 436 | 4407 | 1.059 (0.766 to 1.464) | 1.047 (0.757 to 1.448) |
| High-Yes | 40 | 3836 | reference | reference |
| RERI | | | -0.008 (-0.354 to 0.339) | 0.008 (-0.334 to 0.349) |
| AR | | | -0.005 (-0.256 to 0.245) | 0.006 (-0.245 to 0.256) |
| SI | | | 0.981 (0.402 to 2.396) | 1.022 (0.389 to 2.687) |

**Supplementary Figure S3 Associations of free testosterone and oestradiol with prostate cancer risk (mutual adjustment)**

**AP** – attributable proportion due to interaction; **cases** – number of incident prostate cancer cases per group; **rate** – number of incident prostate cancer cases per 1,000,000 person years of follow-up per group; **CI** – confidence interval; **HR** – hazard ratio; **MI** – multiplicative interaction term of each biomarker individually (z-scores for free testosterone or no/yes for oestradiol) with either BMI (z-scores) or diabetes (no/yes); **NW** – BMI<25 kg/m$^2$; **OW** – BMI≥25 to <30 kg/m$^2$; **Obese** – BMI≥30 kg/m$^2$; **RERI** – relative excess risk from interaction; **SD** – standard deviation; **SHBG** – sex hormone binding globulin; **SI** – synergy index.

**#** – Cox proportional hazards models in men overall with available oestradiol, total and free testosterone, and SHBG measurements (n=156,625), including either free testosterone (z-scores, value minus mean divided by SD, following log-transformation) or oestradiol≥175 pmol/L (no/yes) as exposure, stratified by age at recruitment, region of the assessment centre, and family history of prostate cancer, and adjusted for diabetes status, BMI, ABSI, HI, height (z-scores), recent weight change, smoking status, alcohol consumption, Townsend deprivation index, time of blood collection, and, for free testosterone, also SHBG (z-scores following log-transformation).

**Low/High–No/Yes** – groups of men according to a cross-classification of each biomarker individually (low/high), dichotomised at the median for free testosterone (≥243 pmol/L) or at the lowest detected level for oestradiol (≥175 pmol/L), with either obese or diabetes (no/yes).

## References

**References cited in the main document**:

4. Markozannes G, Tzoulaki I, Karli D, Evangelou E, Ntzani E, Gunter MJ et al. Diet, body size, physical activity and risk of prostate cancer: An umbrella review of the evidence. *Eur J Cancer*. 2016;69:61-9. doi:10.1016/j.ejca.2016.09.026.

6. Christakoudi S, Riboli E, Evangelou E, Tsilidis KK. Associations of body shape phenotypes with sex steroids and their binding proteins in the UK Biobank cohort. *Sci Rep*. 2022;12(1):10774. doi:10.1038/s41598-022-14439-9.

8. Christakoudi S, Tsilidis KK, Evangelou E, Riboli E. A Body Shape Index (ABSI), hip index, and risk of cancer in the UK Biobank cohort. *Cancer Med*. 2021;10(16):5614-28. doi:10.1002/cam4.4097.

17. Christakoudi S, Riboli E, Evangelou E, Tsilidis KK. Associations of body shape index (ABSI) and hip index with liver, metabolic, and inflammatory biomarkers in the UK Biobank cohort. *Sci Rep*. 2022;12(1):8812. doi:10.1038/s41598-022-12284-4.

35. Nagayama D, Krakauer JC, Krakauer NY, Sugiura T, Watanabe Y, Shimizu K et al. Cumulative Cigarette Consumption is Associated with Cardio-Ankle Vascular Index (CAVI) Mediated by Abdominal Obesity Assessed by A Body Shape Index (ABSI): A Cross-Sectional Study. *J Atheroscler Thromb*. 2023. doi:10.5551/jat.64221.

36. Rinkūnienė E, Petrulionytė E, Dženkevičiūtė V, Petrulionienė Ž, Senulytė A, Puronaitė R et al. Prevalence of Cardiovascular Risk Factors in Middle-Aged Lithuanian Men Based on Body Mass Index and Waist Circumference Group Results from the 2006-2016 Lithuanian High Cardiovascular Risk Prevention Program. *Medicina (Kaunas)*. 2022;58(12). doi:10.3390/medicina58121718.

**Supplementary references**:

54. Christakoudi S, Tsilidis KK, Evangelou E, Riboli E. Interactions of platelets with obesity in relation to lung cancer risk in the UK Biobank cohort. *Respir Res*. 2023;24(1):249. doi:10.1186/s12931-023-02561-9.

55. Szulc P. Role of sex steroids hormones in the regulation of bone metabolism in men: Evidence from clinical studies. *Best Pract Res Clin Endocrinol Metab*. 2022;36(2):101624. doi:10.1016/j.beem.2022.101624.

56. Christakoudi S, Pagoni P, Ferrari P, Cross AJ, Tzoulaki I, Muller DC et al. Weight change in middle adulthood and risk of cancer in the European Prospective Investigation into Cancer and Nutrition (EPIC) cohort. *Int J Cancer*. 2021;148(7):1637-51. doi:10.1002/ijc.33339.

57. Zhao J, Leung JYY, Lin SL, Mary Schooling C. Cigarette smoking and testosterone in men and women: A systematic review and meta-analysis of observational studies. *Prev Med*. 2016;85:1-10. doi:10.1016/j.ypmed.2015.12.021.

58. Marom-Haham L, Shulman A. Cigarette smoking and hormones. *Curr Opin Obstet Gynecol*. 2016;28(4):230-5. doi:10.1097/gco.0000000000000283.

59. Jochems SHJ, Fritz J, Häggström C, Järvholm B, Stattin P, Stocks T. Smoking and Risk of Prostate Cancer and Prostate Cancer Death: A Pooled Study. *Eur Urol*. 2023;83(5):422-31. doi:10.1016/j.eururo.2022.03.033.

60. Santi D, Cignarelli A, Baldi M, Sansone A, Spaggiari G, Simoni M et al. The chronic alcohol consumption influences the gonadal axis in men: Results from a meta-analysis. *Andrology*. 2023. doi:10.1111/andr.13526.

61. Hong S, Khil H, Lee DH, Keum N, Giovannucci EL. Alcohol Consumption and the Risk of Prostate Cancer: A Dose-Response Meta-Analysis. *Nutrients*. 2020;12(8). doi:10.3390/nu12082188.

62. D'Andrea S, Spaggiari G, Barbonetti A, Santi D. Endogenous transient doping: physical exercise acutely increases testosterone levels-results from a meta-analysis. *J Endocrinol Invest*. 2020;43(10):1349-71. doi:10.1007/s40618-020-01251-3.

63. Maillard F, Pereira B, Boisseau N. Effect of High-Intensity Interval Training on Total, Abdominal and Visceral Fat Mass: A Meta-Analysis. *Sports Med*. 2018;48(2):269-88. doi:10.1007/s40279-017-0807-y.

64. Blanquet M, Legrand A, Pélissier A, Mourgues C. Socio-economics status and metabolic syndrome: A meta-analysis. *Diabetes Metab Syndr*. 2019;13(3):1805-12. doi:10.1016/j.dsx.2019.04.003.

65. Kilpeläinen TP, Talala K, Raitanen J, Taari K, Kujala P, Tammela TLJ et al. Prostate Cancer and Socioeconomic Status in the Finnish Randomized Study of Screening for Prostate Cancer. *Am J Epidemiol*. 2016;184(10):720-31. doi:10.1093/aje/kww084.

66. Crawford ED, Poage W, Nyhuis A, Price DA, Dowsett SA, Gelwicks S et al. Measurement of testosterone: how important is a morning blood draw? *Curr Med Res Opin*. 2015;31(10):1911-4. doi:10.1185/03007995.2015.1082994.

67. Van de Velde F, Reyns T, Toye K, Fiers T, Kaufman JM, T'Sjoen G et al. The effects of age and obesity on postprandial dynamics of serum testosterone levels in men. *Clin Endocrinol (Oxf)*. 2020;92(3):214-21. doi:10.1111/cen.14141.
